# Impacts of heat stress and storm events on the benthic communities of Kenting National Park (Taiwan)

**DOI:** 10.7717/peerj.11744

**Published:** 2021-07-23

**Authors:** Lauriane Ribas-Deulofeu, Vianney Denis, Pierre-Alexandre Château, Chaolun Allen Chen

**Affiliations:** 1 Biodiversity Research Center, Academia Sinica, Taipei, Taiwan; 2 Taiwan International Graduate Program-Biodiversity, Academia Sinica, Taipei, Taiwan; 3 Department of Life Science, National Taiwan Normal University, Taipei, Taiwan; 4 Institute of Oceanography, National Taiwan University, Taipei, Taiwan; 5 Department of Marine Environment and Engineering, National Sun Yat-sen University, Kaohsiung, Taiwan; 6 Department of Life Science, Tunghai University, Taichung, Taiwan

**Keywords:** Coral bleaching, Typhoon, Community shift, Refuge, Climate change, Coral reef, Taiwan

## Abstract

Over the past few decades, extreme events—such as ocean warming, typhoons, and coral bleaching—have been increasing in intensity and frequency, threatening coral reefs from the physiological to ecosystem level. In the present study, the impacts of rising seawater temperatures, typhoons, and coral bleaching events on benthic communities were seasonally assessed over a 21 month-period, using photo-transects at 11 sites in Kenting National Park (KNP), Taiwan. Between August 2015 and April 2017, seven typhoon events were recorded and* in situ* seawater temperatures in KNP reached a maximum of 31.2 °C, as opposed to an average maximum SST of 28.8 °C (2007–2016). The state and response of benthic communities to these events were interpreted based on the environmental conditions of KNP. The repeated storms lowered the levels of thermal stress during the 2015–2016 El Niño event and may have mitigated its impact on the Taiwanese coral reefs. However, storm-induced local shifts from coral to macro-algae dominance were observed. Storms may mitigate the negative effects of heatwaves, but the mechanical damage induced by the storms may also decrease the structural complexity of reefs and their associated diversity. Eventually, despite reef persistence, the composition and function of remnant communities may profoundly diverge from those in regions with less active storms.

## Introduction

Climate change is transforming our biosphere and jeopardizing the services it provides to humans (Pecl et al., 2017). In the marine realm, increases in seawater temperatures, ocean acidification, and the intensification of storms have already modified past patterns of diversity worldwide (Harley et al., 2006; Lejeusne et al., 2010; Vergés et al., 2014; Hughes et al., 2017a). Coral reefs are among the most valuable marine ecosystems, contributing 35.8 billion US$ to the global economy each year (Spalding et al., 2017) and supporting the livelihoods of millions of people around the world (Teh, Teh & Sumaila, 2013). Over the last four decades, increases in both the frequency and duration of periods of abnormally high seawater temperatures across large geographic areas (i.e., heat waves) have caused more frequent and devastating episodes of coral bleaching (Eakin et al., 2009; Hughes et al., 2018a), i.e., the disruption of the symbiotic relationship that corals maintain with their algal symbionts (Hoegh-Guldberg, 1999). These episodes often transform coral reef communities (Hughes et al., 2018a; Stuart-Smith et al., 2018), compromising the role of scleractinians as ecosystem engineers (Wild et al., 2011).

In 1998, a strong El Niño event triggered the first global mass bleaching event and decimated about 16% of all coral reefs on Earth (Wilkinson & Souter, 2008). A decade later, another warm episode caused a second widespread bleaching event, which led to the bleaching of 80% of the western Atlantic and 60% of the Australasian and Indian Ocean reefs (Hughes et al., 2018a). The incomplete 2014–2015 El Niño formation followed by the strong 2015–2016 El Niño event caused seawater temperature anomalies in the southern and northern hemispheres and triggered a third global mass bleaching event (Eakin, Sweatman & Brainard, 2019), the most extensive and severe ever recorded (Hughes et al., 2017b; Hughes et al., 2018a; Moritz et al., 2018). The reports that followed confirmed that this bleaching event was unprecedented in its geographic scope and intensity, and that it led to 75% of reefs worldwide presenting signs of bleaching (Hughes et al., 2018a); that said, bleaching intensity was variable from region to region (Eakin, Sweatman & Brainard, 2019).

Global losses in reef diversity, functioning, and economics as a result of this third global mass bleaching event are still largely unknown. However, regional studies have already provided insights into its impacts at the local level. On the Great Barrier Reef (GBR), 91.1% of the surveyed reefs presented signs of bleaching in 2016, which resulted in a loss of over half of the living coral cover (Stuart-Smith et al., 2018), while reefs in Moorea (French Polynesia) only suffered minor bleaching (Edmunds, 2017). In the central Red Sea in Saudi Arabia, bleaching affected an average of 2.2 ± 2.7% of the benthic cover in offshore reefs and 53.7 ± 14.6% in inshore ones (Monroe et al., 2018) during the 2015 bleaching event. Bleaching susceptibility decreased with distance from the shore (Monroe et al., 2018) which could be due to the effects of increasing depth and isolation from anthropogenic stressors (Carilli et al., 2009). Unfortunately, by 2017 the Persian Gulf was considerably impacted again by mass-bleaching event, losing 73% of its coral cover within a year, despite being considered as the most thermally tolerant coral region in the world (Burt et al., 2019). Maldivian reefs showed a 75% loss in live coral cover after the 2016 bleaching event (Perry & Morgan, 2017). Regional disparity was further demonstrated by Sully et al. (2019), who examined the effects of latitude and temperature variations on the mitigation of bleaching events. Equatorial reefs appear more resistant to bleaching events than sub-tropical reefs at similar thermal stress levels (possibly due to the combined effects of species composition, genetic diversity, and thermal stress history) (Sully et al., 2019).

Other studies have observed that wind events such as typhoons, cyclones, hurricanes, and other storms induce extensive cooling in seawater temperatures, decreasing heat stress on corals (Manzello et al., 2007; Carrigan & Puotinen, 2014; Doong, Peng & Babanin, 2019; Yang et al., 2019). Indeed, events such as typhoons induce seawater temperature drops via: (1) latent heat flux, as the strong winds lead to evaporative cooling at the sea surface (Paparella et al., 2019); and (2) the powerful waves and currents generated which lead to water column mixing where deeper, cooler water layers decrease shallow water temperatures (Doong, Peng & Babanin, 2019; Yang et al., 2019). Typhoons have been shown to cool waters along their path by as much as 11 °C and decrease heat stress in areas >150,000 km^2^ (Shang et al., 2008; Carrigan & Puotinen, 2014). Reefs can benefit from the presence of a storm as far as 400 km away and its cooling effect can last for as long as one month (Manzello et al., 2007). Therefore, if thermal anomalies and storm seasons are synchronous, regions along typhoon tracks may be less likely to experience widespread bleaching (Manzello et al., 2007; Shang et al., 2008). In 1998, the cyclone Anacelle prevented a major heat stress event and preserved the Mauritius reefs from extensive bleaching (Turner et al., 2000). In the Caribbean, hurricanes cooled waters and reduced the impacts of the 2005 and 2010 temperature anomalies (Carrigan & Puotinen, 2014). Nevertheless, swells and waves from typhoons still cause mechanical damage to reefs that can also have consequences for ecosystem functioning (Harmelin-Vivien, 1994; White et al., 2013; White et al., 2017; Puotinen et al., 2020). In other regions such as the Persian Gulf, studies have shown that windy events (>4 m s^−1^) can be sufficient to control the occurrence and severity of bleaching, via latent heat flux through wind-induced surface evaporation (Paparella et al., 2019). They showed strong correlation between daily temperature change and wind speed, with seawater temperature drops becoming more pronounced as wind intensifies (Paparella et al., 2019; Burt et al., 2019).

Taiwan is a large continental island that faces the Pacific Ocean and, on average, is hit by 4.5 typhoons annually (Lee et al., 2006). From 1998 to 2017, 108 typhoons were recorded in Taiwan (Doong, Peng & Babanin, 2019), and their frequency has increased in recent decades (Tu, Chou & Chu, 2009). The waters surrounding Taiwan are among the most rapidly warming in the world (Belkin, 2009; Toda & Watanabe, 2020), pushing corals closer to their upper thermal limits. Widespread bleaching events associated with El Niño events were reported in 1998 and 2007 throughout Kenting National Park (KNP) (Dai et al., 1999; Goreau et al., 2000; Tung et al., 2007). The 2010 bleaching event, however, only moderately affected reefs in Taiwan (Tew et al., 2014).

For the 2007–2016 period, monthly summer seawater temperature recorded by the Central Weather Bureau (CWB, http://www.cwb.gov.tw/eng/) in KNP averaged 28.6 °C, 28.8 °C and 28.6 °C for June, July and August respectively, and the maximum seawater temperature recorded was 31.7 °C (August 2011). During the 2015–2016 El Niño event, seawater temperature anomalies were observed during summer seasons in southern Taiwan, reaching a maximum of 31.2 °C (August 2016, Kenting). The region was repeatedly placed under bleaching alert (Level 1 and 2) by the National Oceanic and Atmospheric Administration (NOAA) Coral Reef Watch (Liu et al., 2014). Recreational divers observed local bleaching in shallow waters; this was relayed by KNP authorities, and a global study categorized the event as severe in Taiwan (Hughes et al., 2018a). However, no quantitative data supported this assertion. During each of the studied years, successive typhoons seem to have attenuated the heat stress (and subsequent bleaching) by cooling down seawaters directly along the typhoons’ tracks.

In the present study, we documented the responses of the benthic communities throughout KNP during the 2015–2016 El Niño event. We hypothesized that the storms’ synchronicities with heatwaves during our survey period offered the reefs temporary relief from heat stress, limiting the extent of the bleaching event. We discuss the contrasting roles that typhoons and storms could play in local reef persistence in the context of global ocean warming, along with the associated risk of coral diversity loss, which could lead to a widespread deterioration of benthic communities in KNP.

## Materials and Methods

### Study area

**Figure 1:**
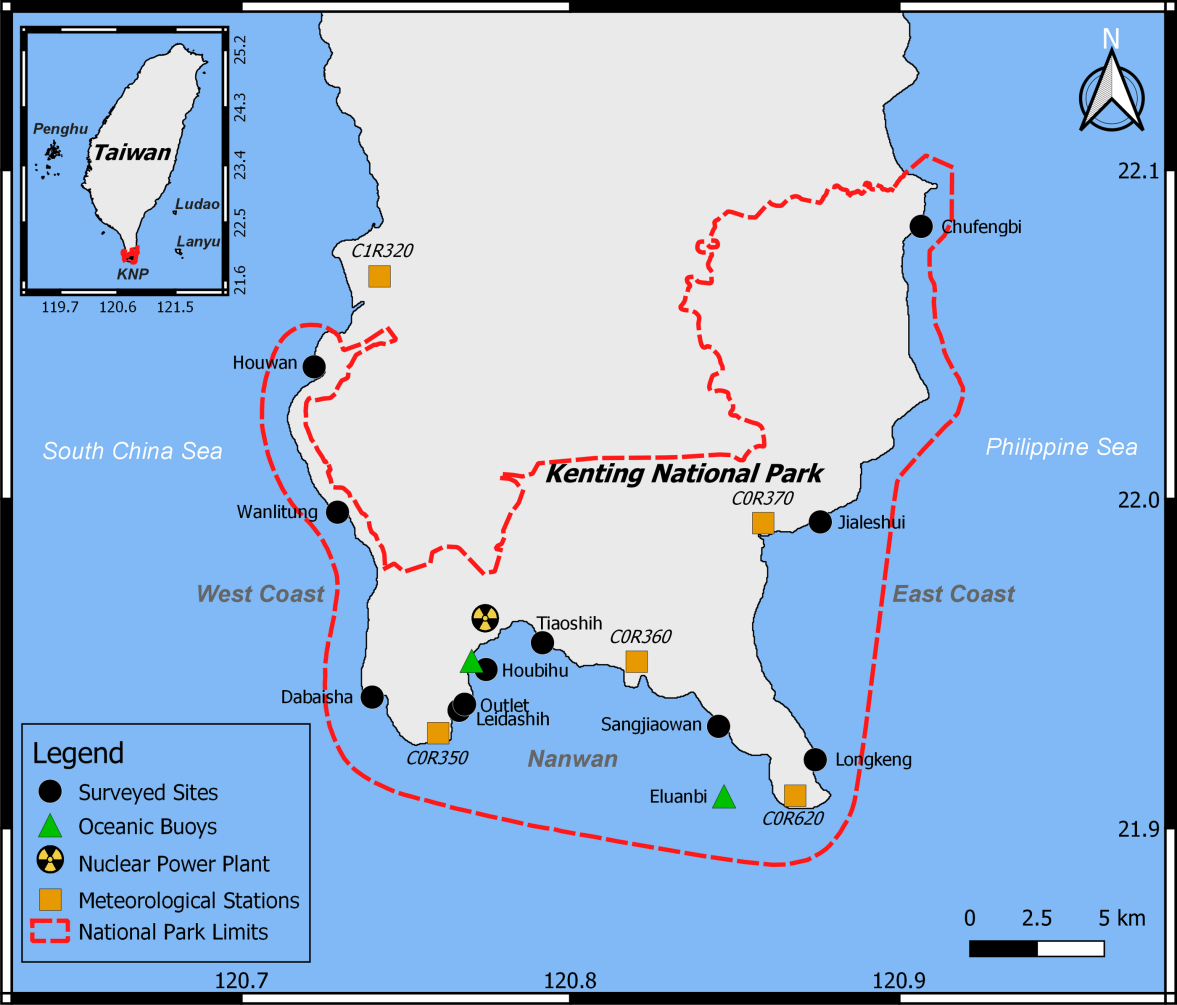
Study area. Within KNP (dashed red line), 11 reefs were surveyed (black circles), oceanic data were collected from two buoys (green triangles), and meteorological information from five stations were combined (orange squares).

This study targeted the Hengchun Peninsula (21.90°N, 120.79°E), located at the southernmost tip of the main island of Taiwan (Fig. 1). KNP was established there in 1985 as the first national park in Taiwan, it encompasses both terrestrial and marine sectors, including many fringing reefs that shape the coast at depths <30 m (Denis et al., 2019). KNP harbors a high level of marine diversity (Dai & Horng, 2009a). The high frequency of tropical storms (Dai, 1991) have previously caused severe damage to the marine ecosystems in the region (Kuo et al., 2011; Kuo et al., 2012). Typhoons and tropical storms usually hit southern Taiwan from the southeast to northwest, so the west side of the semi-enclosed bay of Nanwan, the central part of KNP, is usually the most exposed area to storm damage (Dai, 1991). On the other hand, the eastern side of the bay and the west coast of Hengchun Peninsula are less exposed due to the topography of the region (Dai, 1991). Additionally, two types of monsoons influence the region: the northeastern monsoon induces dryer weather but stronger winds from November to April, while the southern monsoon induces heavy rains, usually accompanied by strong but short windy episodes, from May to September (Dai, 1991). In fact, the southern monsoon accounts for 90% of the annual precipitation in the region. Storms induced by this southern monsoon can have similar effects to typhoon events: strong winds, runoff, exacerbated vertical mixing of seawater, and an increase in the magnitude of daily temperature drops (Jan & Chen, 2009; Rivest & Gouhier, 2015). In the bay of Nanwan, a tidal upwelling can cause further sudden temperature drops as the topography of the bay induces the formation of cyclonic eddies. These eddies move from west to east throughout the bay, during the ebb tidal current, mixing the cooler deeper seawater layers with the warmer shallow ones (Lee et al., 1997; Lee et al., 1999; Hsu et al., 2020). The upwelling within Nanwan has been shown to play an important role in the physiology of marine organisms in the area (Chen et al., 2005; Keshavmurthy et al., 2019), especially during summer periods, when the upwelling can cause daily variations in seawater temperatures to increase by up to 2.5 °C (Hsu et al., 2020). In addition, some sites in Nanwan have been further affected by warmer waters (as much as 2 to 3 °C higher than other sites in summer) from the nearby outlet of the nuclear power plant since its opening in 1987 (Huang, Hung & Fan, 1991; Carballo-Bolaños et al., 2019). Over the past decade (2009–2018), economic development has increased the number of tourists in the region from 3.5 million to over 8 million, with human activities peaking in summer (Maynard et al., 2019). Land-based pollution (Meng et al., 2008; Liu et al., 2012; Jiang et al., 2015) and overfishing (Liu et al., 2009) were previously hypothesized to conjointly impact both the heterogeneity observed in the structure of the benthic communities (Ribas-Deulofeu et al., 2016) and the benthic diversity of KNP (Lin & Denis, 2019).

Previous studies reported an important spatial heterogeneity within the KNP benthic community, with higher algal cover closer to river estuaries (Meng et al., 2008). The benthic communities of the western coast of KNP as well as Nanwan Bay are heavily impacted by anthropogenic disturbances such as coastal development, agriculture, and tourism (Meng et al., 2008; Liu et al., 2012; Ribas-Deulofeu et al., 2016). Within Nanwan Bay, disparity in the benthic community has also been reported: the western side of the bay has greater coral cover than the eastern side since it is more sheltered from land-based water pollution and urbanization due to the presence of the nuclear power plant (Ribas-Deulofeu et al., 2016). Accordingly, eleven sites were selected to represent this spatial disparity (Fig. 1): three sites on the west coast of Hengchun Peninsula (Houwan, Wanlitung, and Dabaisha), three on the east coast(Longkeng, Jialeshui, and Chufengbi), and five in the bay of Nanwan (Leidashih, Outlet, Houbihu, Tiaoshih, and Sangjiaowan). Each site was investigated at a depth of 5 m. Five assessments of the benthic communities were performed between 2015 and 2017: August 2015, April 2016, September 2016, October 2016, and April 2017. Bleaching was expected to be observed in August 2015, as well as in September and October 2016. However, due to weather conditions, neither west nor east coasts could be accessed in October 2016, and the east coast could not be accessed in September 2016.

### Biotic surveys

Following the method described in Ribas-Deulofeu et al. (2016), the benthic communities were surveyed along five 20 m long photo-transects, and a total of 105 photographs (0.5 m x 0.5 m) were captured at each site during each survey. In the initial survey and for each site, a location at a depth of 5 m was marked using a metal rod. It was used as our starting point here, and in the later surveys, to lay five transects haphazardly positioned following the reef contour in a given direction. Photographs were analyzed using 50 random points overlaid on each picture using the CPCe v4.0 software (Kohler & Gill, 2006), and benthic organisms were identified using 133 Operational Taxonomic Units (OTUs). Scleractinian (89 OTUs) were identified at the genus level, using Veron (2000), Dai & Horng (2009a) and Dai & Horng (2009b), and categorized into a morpho-functional group (hereafter morphotype) (branching, foliose, encrusting, massive, or tabulate). The algae were divided into 11 OTUs: three macrophyte OTUs (green, red, and brown), six filamentous OTUs (brown, green, red, Cyanophyta, turf, and turf sedimented) and two calcareous OTUs (Encrusting Coralline Algae [ECA] and articulated calcareous algae) (Titlyanov & Titlyanov, 2012). Octocorals (10 OTUs) were identified at the genus level (Fabricius & Alderslade, 2001) and associated with a morphotype (clustered, columnar, bushy, digitate, lobate, or massive) following Lin & Denis (2019). Data was later summarized into 14 major categories (Ascidian, Encrusting Coralline Algae, Hydrozoan, Macro-algae, Octocoral, Scleractinian, Sea Anemone, Sponge, Turf, Zoantharian, Other Life, Unknown, Bare, and Unstable Substrate). Symbiodiniaceae-associated organisms were also divided in three bleaching categories: “no obvious sign of bleaching”, “sign of discoloration”, and “totally bleached”. To estimate the extent of the bleaching event, we retained the “totally bleached” observations for each OTU at the regional scale. 104 OTUs were identified as being associated with Symbiodiniaceae with bleaching potential: 89 scleractinian OTUs, 10 octocoral OTUs, two hydrozoan OTUs, two zoantharian OTUs, and one sea anemone OTU.

### Abiotic surveys

#### Storm indicators: rainfall, wind speed, and wave height

Wave height data from the Eluanbi buoy (21.9006°N, 120.8314°E) and hourly rainfall along with additional wind speed data from five meteorological stations within KNP (Table S1, Fig. 1) were used to detect storm events, such as typhoons. Rainfall datasets were converted into daily-cumulative rainfall per station and then averaged throughout KNP, while wind speeds from the five terrestrial stations were averaged by day. Wave height, rainfall and wind data were provided by the Central Weather Bureau (CWB) and the Water Resources Agency of Taiwan (Table S1) and, covered the period from our first (August 2, 2015) to last (April 24, 2017) survey.

#### Seawater temperature and anomalies

Seawater temperatures were recorded at three surveyed sites: Houwan (west coast), Jialeshui (east coast), and Houbihu (Nanwan). One HOBO pendant temperature data logger (UA-002-64) per site was affixed to the substrate at 5 m in depth and took measurements every 30 min. However, the logger at Houbihu malfunctioned and stopped recording after October 2016. These datasets were used to track **in situ** temperature experienced by reef organisms in KNP during the 2015–2016 El Niño event. To illustrate the cooling effect of typhoons, background seawater temperatures were calculated for each of the three sites as the average seawater temperature over the seven days prior to a typhoon, following Doong, Peng & Babanin (2019). For each site, maximum seawater temperature drops induced by each typhoon were calculated as the difference between the lowest local daily seawater temperature during each typhoon and their respective pre-typhoon background seawater temperature. We reported in Table 1 the seawater temperature drops induced by each typhoon as the average of the maximum seawater temperature drops calculated for Houwan, Jialeshui and Houbihu.

**Table 1:** Typhoons in the vicinity of Taiwan from August 2015 to April 2017. Information related to typhoon trajectories, and intensities along with the minimum sea level pressure (hPa), maximum wind speed (m/s), and maximum gust (m/s) recorded in KNP by the CWB during the respective typhoons. The decreases in average SST induced by each typhoon were calculated based on Doong, Peng & Babanin (2019).

Typhoon	Starting day	Ending day	Center of the landing area	Category	Minimum Sea Level Pressure (hPa)	Maximum Wind Speed (m/s)	Maximum Gust (m/s)	Averaged max SST drop during typhoon (°C)	Note
Soudelor	8∕6∕2015	8∕9∕2015	Central Taiwan	3	979.1	16	34.9	−0.56 ± 0.50	
Goni	8∕20∕2015	8∕23∕2015	Not landed	1	992.8	10	19	+0.3 ± 1.48	
Dujuan	9∕27∕2015	9∕29∕2015	Northern Taiwan	4	992.8	11.2	25.5	−1.69 ± 0.79	
Nepartak	7∕6∕2016	7∕9∕2016	Southern Taiwan	5 (super-typhoon)	985.4	11.3	26.1	−0.91 ± 0.42	
Meranti	9∕12∕2016	9∕15∕2016	South-Western Taiwan	5 (super-typhoon)	963.7	24.7	52.2	−0.38 ± 0.67	
Malakas	9∕15∕2016	9∕18∕2016	Not landed	4	1002.1	6.3	13.4	−1.13 ± 0.46	Overlapped with typhoon Meranti
Megi	9∕25∕2016	9∕28∕2016	Central Taiwan	4	984	15.9	31.3	+0.06 ± 0.08	

Degree Heating Weeks (DHW) were further calculated to assess the cumulative effect of thermal stress on coral reef organisms (Montgomery & Strong, 1994; Liu, Strong & Skirving, 2003; Liu et al., 2014). DHW represents the accumulation of temperature anomalies that are equal to or higher than the local bleaching threshold over a consecutive 12-week period at a given location (Liu, Strong & Skirving, 2003). KNP’s Maximum Monthly Mean temperature (MMM) was calculated from daily average seawater temperatures recorded by the CWB buoy near Houbihu Harbor (Fig. 1) from January 2007 to December 2014 (Table S1). Once MMM was determined, the local bleaching threshold was established as the MMM for KNP plus 1 °C (Goreau & Hayes, 1994; Strong, Barrientos & Duda, 1996). Thermal stress was calculated locally by subtracting this threshold from the daily **in situ** maximum seawater temperatures at Houbihu, Houwan, and Jialeshui. Weekly averages of thermal stress were calculated for the 12 successive weeks prior to each day from November 2015 to April 2017 to obtain the final local Degree Heating Week (DHW_L_). This was compared to the regional estimation from the NOAA Coral Reef Watch (Liu et al., 2014) (DHW_R_) from the nearby virtual station (22.875°N, 120.55°E). The NOAA Coral Reef Watch has four levels of bleaching alerts based on the DHW levels: (a) Bleaching Watch, when DHW is 0–1 °C weeks; (b) Bleaching Warning, when DHW is 1–4 °C weeks; (c) Bleaching Alert Level 1, when DHW is 4–8 °C weeks; and (d) Bleaching Alert Level 2, when DHW>8 °C weeks (Liu et al., 2014).

### Data analyses

Average percentage and Standard Deviation (SD) of OTUs cover, bleaching occurrence, and the contribution of each genus to the observed bleaching were calculated. Changes in major categories were tested at the scale of KNP from our first to last survey using the non-parametric two sample sign test. The OTU richness and the *α*-diversity were estimated for each area in KNP (Oksanen, 2018). In addition, for each region, changes in diversity (OTU richness and Shannon diversity) were examined between surveys (Hsieh, Ma & Chao, 2016). Multivariate analyses were performed on arcsine transformed dataset, where OTUs that appeared in < 5% of the transects were excluded from multivariate analyses (McCune & Grace, 2002) to minimize the effect of abundant OTUs and OTUs with numerous zeros (Borcard, Gillet & Legendre, 2018). Significant changes in benthic communities between KNP’s areas and between surveys were identified using permutational multivariate analysis of variance (ADONIS). ADONIS values were generated using Bray–Curtis distance matrices from 9,999 random permutations. *Post-hoc* pairwise permutational multivariate analyses of variance were performed (Hervé, 2019). Kruskal-Wallis (KW-test) and Dunn tests (Dinno & Dinno, 2017) were used for each area to identify temporal changes in major categories and scleractinian morphological groups contributing to more than 5% of the benthic communities in at least one site. Differences with *p*-values <0.05 were considered significant. Statistical analyses on biodiversity, abiotic, and other factors were performed in R(v3.5.1) using the packages vegan (Oksanen, 2018), iNEXT (Hsieh, Ma & Chao, 2016), RVAidememoire (Hervé, 2019), BSDA (Arnholt & Evans, 2017), and dunn.test (Dinno & Dinno, 2017).

## Results

### Abiotic patterns

#### Seawater temperature pattern in KNP

**Figure 2:**
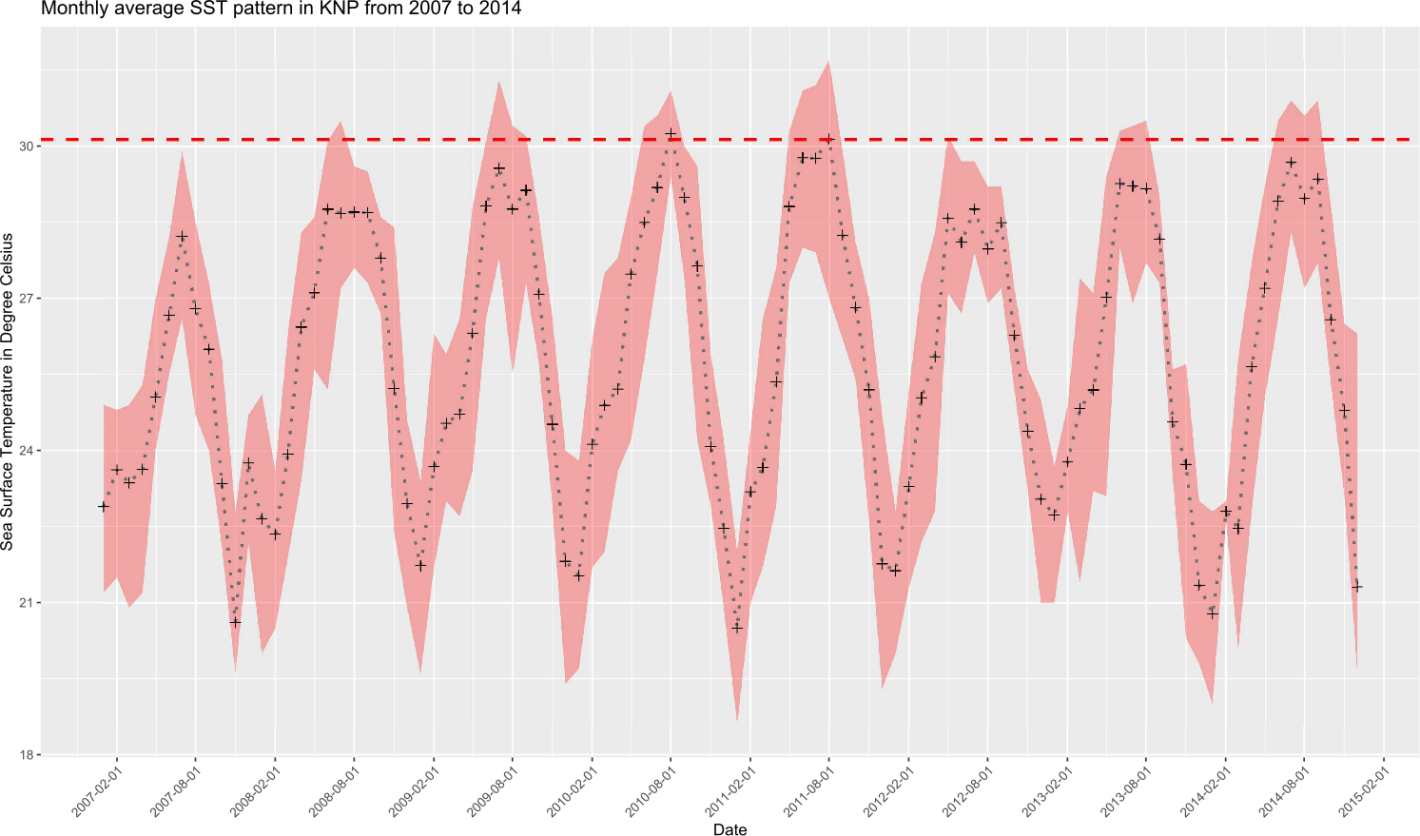
Sea Surface Temperatures (SSTs) in KNP (2007–2014). Monthly average SSTs (black crosses) with monthly maxima and minima (red shaded area). Dashed horizontal red line represents the local bleaching threshold of 30.1 °C calculated in this study (Data: Central Weather Bureau, Taiwan).

**Figure 3:**
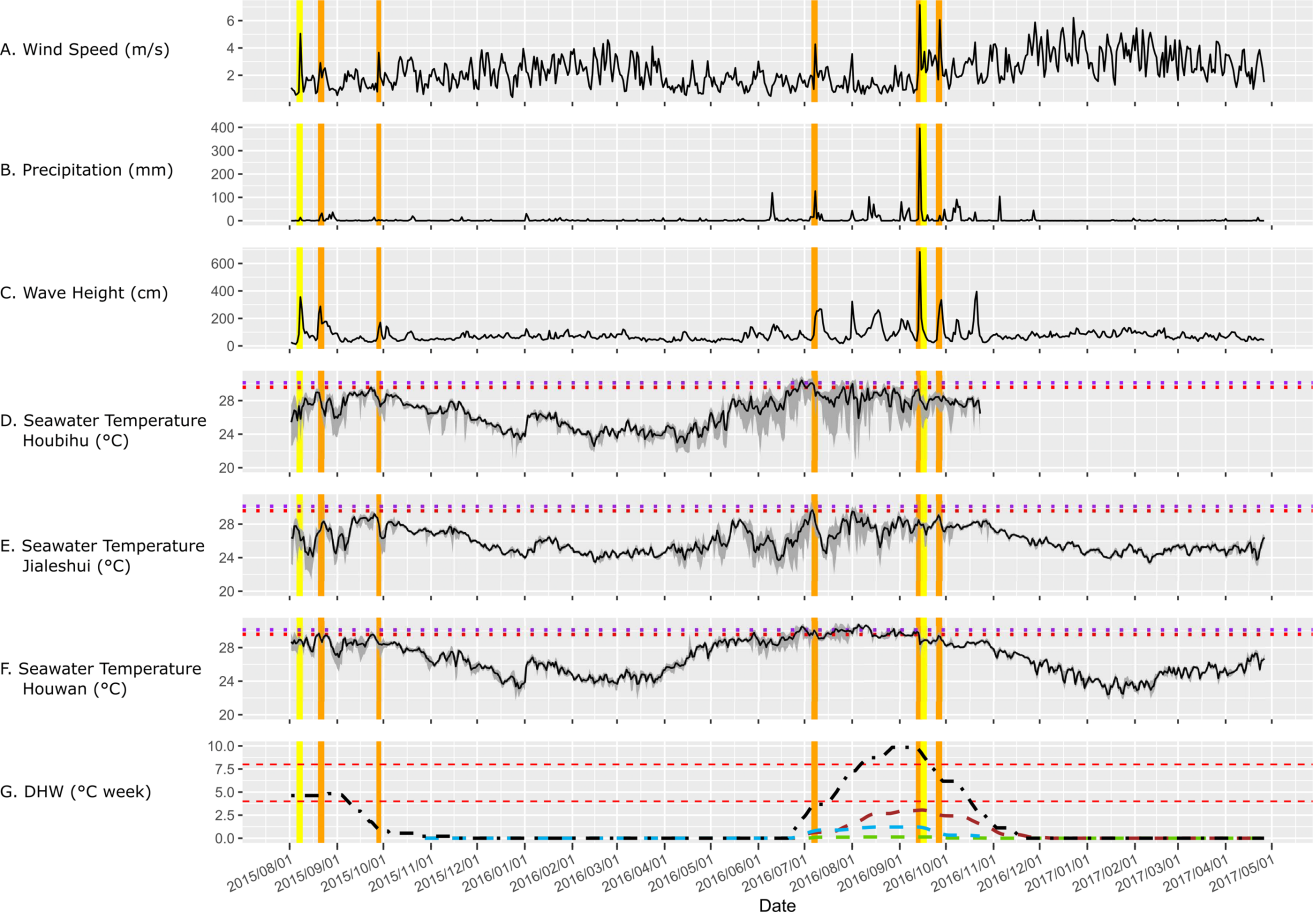
Environmental conditions in KNP (August 2015–April 2017). (A) Average daily wind speed (m/s) and (B) daily rainfall (mm) from the meteorological stations of Taiwan’s Central Weather Bureau (CWB). (C) Wave height (cm) from Eluanbi buoy (Water Resources Agency). (D, E, and F) The average daily seawater temperature at 5m in depth in Houbihu (Nanwan), Jialeshui (east coast), and Houwan (west coast), recorded with HOBO pendant temperature data loggers (UA-002-64). The gray shaded areas represent the range between daily minimum and maximum at each site. Horizontal purple and red dotted lines represent the local bleaching threshold for KNP (30.1 °C) and the bleaching threshold estimated by NOAA for southern Taiwan, respectively. (G) Degree Heating Weeks (DHW_*L*_) from Houbihu (blue dashed line), Jialeshui (green solid line), and Houwan (brown dashed line), and DHW_*R*_ from NOAA for southern Taiwan (black dashed line). The horizontal red dashed lines represent the Bleaching Alert Level 1 threshold (DHW 4 °C weeks) and the Level 2 threshold (DHW 8 °C weeks). Vertical lines represent the typhoons; orange: the ones that landed in Taiwan, and yellow: the ones that did not land on the island but affected its surrounding seas.

Monthly average seawater temperatures over the 2007***–***2014 period ranged from 21.8 °C (January) to 29.1 °C (July, MMM) for the reefs in KNP, leading to a predicted local bleaching threshold of 30.1 °C (Houbihu buoy; Fig. 2), while the bleaching threshold estimated by NOAA (Liu et al., 2014) for southern Taiwan was 29.6 °C (Fig. 3). *In situ* observations during our survey period showed a minimum daily average temperature of 22.4 °C (January 2017) and a maximum of 30.8 °C (August 2016) (Fig. 3) on the west coast (Houwan). On the east coast (Jialeshui), these were 23.4 °C (February 2017) and 29.7 °C (July 2016), respectively (Fig. 3); in Nanwan (Houbihu), these were 22.6 °C (February 2016) and 30.4 °C (June 2016), respectively. Overall, between the coldest and the hottest daily *in situ* seawater temperature recorded, study sites showed an amplitude of 7.8 °C in Houbihu (August 2015–October 2016), 8.4 °C in Houwan, and 6.3 °C in Jialeshui (August 2015–April 2017; Fig. 3).

#### Storm impacts on abiotic parameters

Five typhoons hit Taiwan from August 2015 to April 2017 (orange bars in Fig. 3, Table 1). Two additional typhoons in the vicinity of the island induced heavy rains and strong winds in the surveyed region (yellow bars in Fig. 3, Table 1). Drops in seawater temperature were recorded during five of the seven typhoons (Table 1). Typhoons Goni (+0.3 ± 1.48 °C for August 20–23, 2015) and Megi (+0.06 ± 0.08 °C for September 25–28, 2016) did not induce seawater temperature drops. However, Typhoons Souledor, Meranti, and Malakas caused drops in seawater temperatures throughout KNP (averaged seawater temperature drops: −0.56 ± 0.50 °C for August 6–9, 2015; −0.38 ± 0.67 °C for September 12–15, 2016; and −1.13 ± 0.46 °C for September 15–18, 2016, respectively). The maximum seawater temperature dropped over 0.90 °C on average during Typhoons Dujuan (−1.69 ± 0.79 °C for September 27–29, 2015) and Nepartak (−0.91 ± 0.42 °C for July 6–9, 2016).

In addition, monsoon characteristics clearly differed between seasons, with summers having strong rainy and windy episodes accompanied by higher wave heights and winters being dry and windy. Several local storms occurred during the 2016 southern monsoon season (Fig. 3) and were characterized by their wind speeds (≥4 m s^−1^), precipitation peaks (≥50 mm), and/or high wave heights (≥150 cm). Those peaks were associated with abrupt seawater temperature decreases (the greatest observed drop in average daily seawater temperature between two consecutive days was −2.18 °C at Jialeshui on July 31, 2016, Fig. 3).

#### Heat stress during the 2015–2016 El Niñ o event

DHW_R_ in southern Taiwan rose slightly over Bleaching Alert Level 1 from August 2 to September 7, 2015 (Fig. 3). DHW_R_ rose again over the Bleaching Alert Level 1 threshold on July 18, 2016 and reached Level 2 on August 7, 2016. DHW_R_ peaked (9.85 °C weeks) on August 27 and remained at this level until September 12, 2016, when the super typhoon Meranti hit Taiwan. After Meranti arrived, DHW_R_ started to decrease steadily as successive typhoons hit Taiwan (Fig. 3). When *in situ* seawater temperatures are considered in KNP, DHW_L_ never exceeded the 4 °C weeks threshold during the August 2015–December 2016 period. Indeed, DHW_L_ increased in Houbihu and Houwan starting in late June 2016, peaking at 1.2 °C weeks (August 2016) and 3.1 °C weeks (September 2016), respectively. In Jialeshui, DHW_L_ only started in mid-July, and quickly peaked at 0.1 °C weeks, where it remained until late September 2016.

### Biotic pattern

#### Diversity

**Figure 4:**
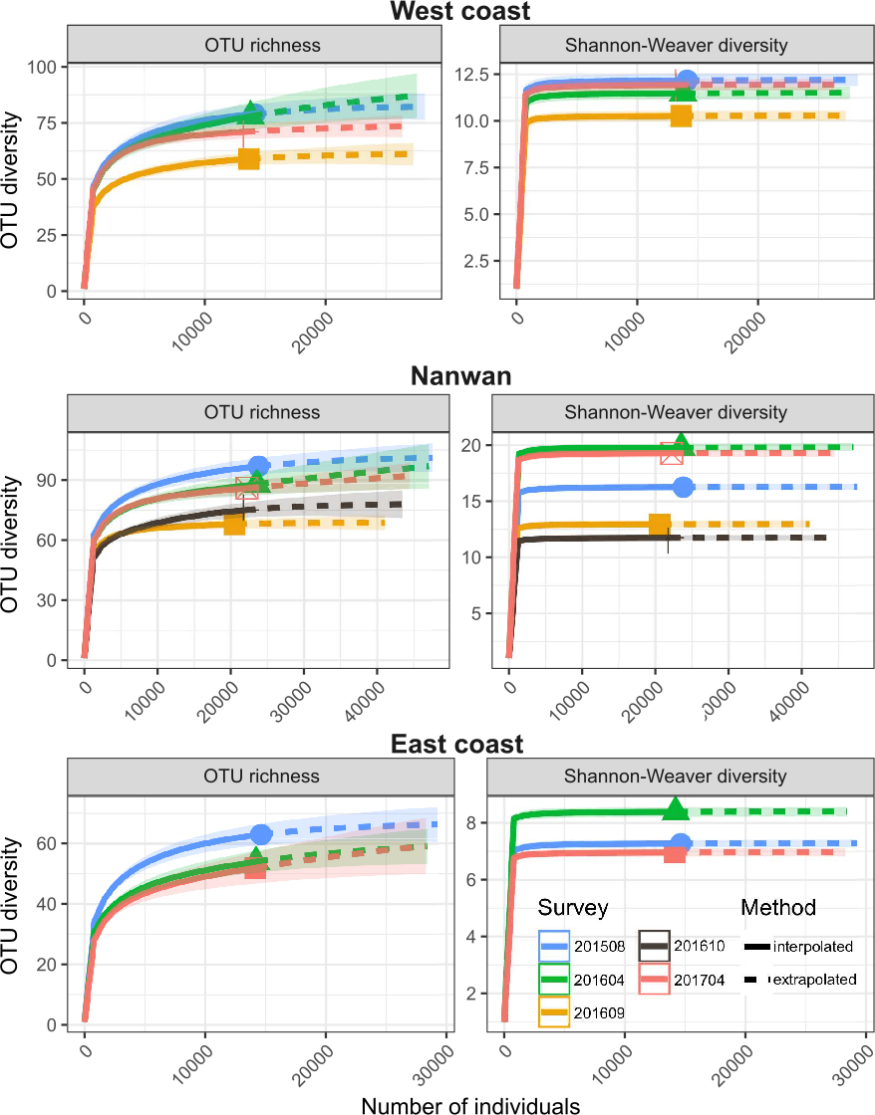
Temporal and spatial distribution of benthic diversity in KNP (August 2015–April 2017). Seasonal iNEXT observed rarefaction and extrapolation curves of OTU richness and Shannon-Weaver diversity for the east and west coasts and Nanwan. The solid lines represent the observed accumulation, dashed lines are the extrapolated accumulation, and shaded areas are the 95% confidence intervals (Hsieh, Ma & Chao, 2016).

During the August 2015–April 2017 period, the OTU richness was higher in Nanwan (121) than in the west coast (103) or east coast (80) (Table 2); similarly, their *α*-OTU richness values were 50.4 ± 9.2, 48.1 ± 5.5, and 33.2 ± 13.1, respectively (Table 2). The three areas of KNP presented 96, 82 and, 59 OTUs with bleaching potential, respectively (Table 2). Diversity indices (OTU richness and Shannon diversity) were higher prior to the 2016 typhoon season and lower during the 2016 typhoon season (September and October 2016) on the west coast and in Nanwan (Fig. 4). Six months after the last 2016 typhoon, benthic communities in those areas showed signs of recovery in terms of OTU richness and abundance, as both OTU richness and Shannon-Weaver diversity returned to levels only slightly lower to those in August 2015 or April 2016 (Fig. 4). However, between August 2015 and April 2017, we witnessed a reduction of the scleractinian OTUs richness of 13% in KNP (respectively, 77 versus 67 scleractinian OTUs; Table 3). Despite a reduced scleractinian OTUs richness observed by the end of our study, recovery trends were observed, as best shown in Nanwan and the west coast (Table 3). In Nanwan, we observed a loss of 27.9% of the scleractinian OTUs richness between our survey in August 2015 (68 scleractinian OTUs) and our post-typhoon survey in September 2016 (49 scleractinian OTUs; lowest Scleractinian OTUs richness observed) but nine scleractinian OTUs were regained between September 2016 and April 2017 (58 scleractinian OTUs), which limited the overall loss of scleractinian OTUs richness in Nanwan to 14.7% during our study period (Table 3). Similarly, on the west coast, scleractinian OTUs richness suffered a reduction of 25.0% between August 2015 (56 scleractinian OTUs) and September 2016 (42 scleractinian OTUs) but regained 10 scleractinian OTUs by April 2017 (52 scleractinian OTUs), limiting the scleractinian OTUs richness loss to 7.14% by the end of our study (Table 3).

**Table 2:** Distribution of diversity and bleached OTUs within KNP (August 2015–April 2017).

**KNP areas**	**OTU diversity**				**Number of bleached OTUs**	
	**OTU Richness (133)**	***α*-diversity**	**Minimum OTUs Richness recorded/site**	**Maximum OTUs Richness recorded/site**	**OTUs with bleaching potential (104)**	**OTUs that bleached at least once (38)**
**East Coast**	80	33.2 ± 13.1	14 *Chufengbi; April 2017*	47 *(Longkeng; August 2015)*	59	4
**Nanwan**	121	50.4 ± 9.2	31 *(Leidashih; October 2016)*	68 *(Tiaoshih; August 2015)*	96	33
**West Coast**	103	48.1 ± 5.5	39 *(Dabaisha; September 2016)*	59 *(Houwan; April 2016)*	82	19

#### Benthic dynamics

**Figure 5:**
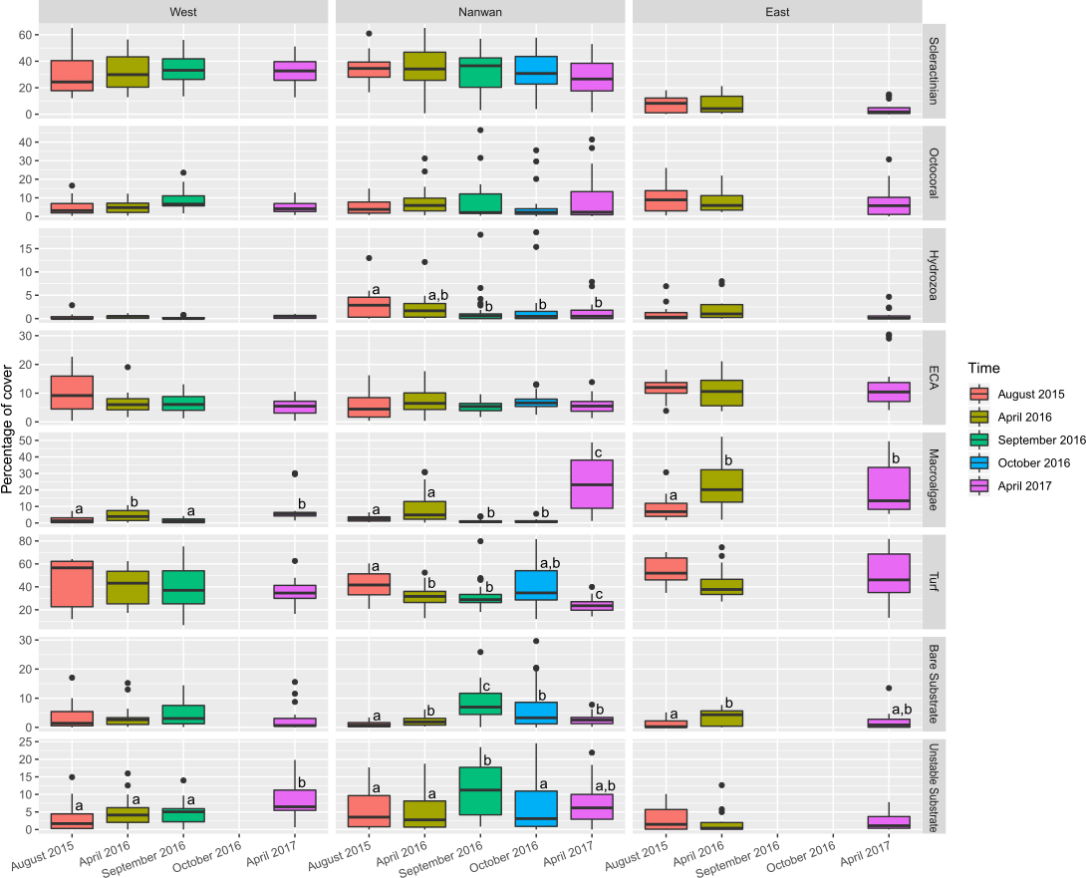
Temporal changes within KNP benthic communities (August 2015–April 2017). Boxplots of eight major categories cover on the east and west coasts of KNP and Nanwan. Superscript letters indicate significant temporal differences (*p* ≤ 0.05) for each major category in the different areas of KNP identified by the Dunn test.

**Figure 6:**
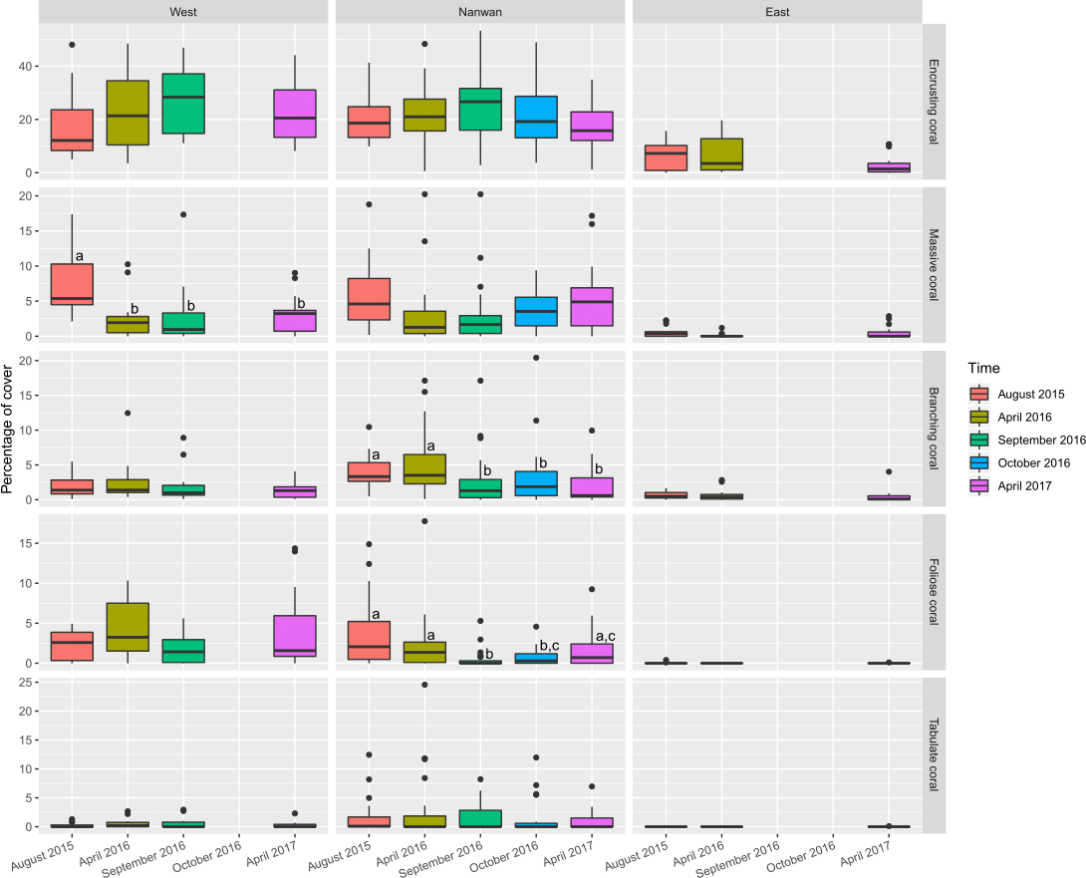
Temporal changes in scleractinians morphological composition within KNP (August 2015–April 2017). Boxplots of scleractinians morphological group cover on the east and west coasts of KNP and Nanwan. Superscript letters represent significance differences (Dunn test, *p* ≤ 0.05) between surveys for the concerned morphotypes.

The benthic communities of KNP showed significant difference between surveys (*F*_(4,225) _ = 6.5, *p* < 0.001, ADONIS, pairwise permutational manova have *p* < 0.05 for all the temporal combinations) but remained dominated by turf algae throughout our study period (average from 2015 to 2017: 38.3 ± 14.2%; Fig. 5, Table S2). During this period, macro-algae cover increased by 60% in a single year (average in April 2016: 11.4 ± 12.1%, average in April 2017: 18.3 ± 13.3%, two-sided two sample sign test *S* = 17, *p* < 0.01). The amount of unstable substrate also increased during our survey period (two-sided two sample sign test *S* = 18, *p* = 0.01; Table S2). A slight decrease in scleractinian cover was observed in April 2017, but it was not significant (27.1 ± 18.2% in April 2016 and 22.1 ± 16.4% in April 2017, two-sided two sample sign test S = 34, *p* = 0.1). During our survey period, the coral assemblages within KNP were dominated by encrusting coral (18.4 ± 11.2%; Fig. 6). Other coral morphotypes were scarcer, particularly the foliose and tabulate species (1.8 ± 2.3% and 1.0 ± 2.2%, respectively; Fig. 6, Table S3). In addition, branching coral cover decreased markedly by 45.6% over two years, from 2.56 ± .01% in August 2015 to 3.27 ± 3.32% in April 2016 and 1.39 ± 1.65% in April 2017 (Fig. 6, Table S3). Octocorals across the three areas remained stable (6.4 ± 4.8% in 2015 and 7.4 ± 9.6% in 2017, two-sided two sample sign test *S* = 32, *p* = 0.28).

**Table 3:** OTUs richness of scleractinians, octocorals, algae and other life categories between surveys (August 2015–April 2017).

Survey	West Coast				Nanwan bay				East Coast				Kenting National Park			
	Scleractinians	Octocorals	Macro-algae	Other Life	Scleractinians	Octocorals	Macro-algae	Other Life	Scleractinians	Octocorals	Macro-algae	Other Life	Scleractinians	Octocorals	Macro-alga	Other Life
August 2015	56	7	3	6	68	9	6	5	40	7	4	7	77	9	7	10
April 2016	53	7	6	4	59	9	6	6	30	7	6	5	70	10	8	8
September 2016	42	5	2	3	49	8	2	3	NA	NA	NA	NA	NA	NA	NA	NA
October 2016	NA	NA	NA	NA	54	8	4	3	NA	NA	NA	NA	NA	NA	NA	NA
April 2017	52	5	5	2	58	8	8	5	31	6	4	4	67	8	7	7

Benthic communities in Nanwan and both the east and west coasts differed significantly (*F*_(2,227)_ = 22.0, *p* < 0.001, ADONIS). Multivariate analysis did not show significant changes in benthic communities throughout our survey period for the east coast (*F*_(2,42)_ = 1.8, *p* = 0.11, ADONIS), but it did highlight significant changes in the west coast and Nanwan (*F*_(3,56) _ = 2.8, *p* < 0.001 and *F*_(4,120)_ = 5.1, *p*< 0.001, ADONIS, respectively; Fig. 5, Tables S2 and S3).

On the east coast, macro-algae cover showed an important increase between surveys (*p*< 0.01, KW-test; Fig. 5) while bare substrate significantly increased in April 2016 and decreased by April 2017 (*p*<0.05, KW-test; Fig. 5). On the west coast, macro-algae showed significant seasonal differences between surveys, with lower covers during summer seasons (August 2015 and September 2016, *p*< 0.001, KW-test; Fig. 5, Table S2), while unstable substrate cover showed a marked increase between September 2016 and April 2017 (*p*< 0.05, KW-test; Fig. 5, Table S2). In addition, when the different scleractinian morphotypes were tested separately, there was a significant decline in massive coral cover on the west coast following our initial survey in August 2015 (*p*<0.001, KW-test, Dunn test; Fig. 6).

Nanwan’s benthic communities presented a significant increase in bare and unstable substrates when typhoons started to hit Taiwan in 2016 (*p*<0.001 and *p*<0.01, respectively, KW-test; Fig. 5, Table S2); macro-algae cover dropped during this period, but increased considerably in April 2017 (*p*< 0.001, KW-test, Dunn test; Fig. 5, Table S2). There was no significant trend in scleractinian cover (*p* = 0.32, KW-test). However, when coral morphotypes were investigated individually, significant temporal patterns were found in massive, branching, and foliose corals (*p*<0.01, *p*< 0.001, and *p*< 0.001, respectively, KW-test; Fig. 6, Table S3). The decline in branching coral cover was particularly stark in Tiaoshih (−83.9%), Sangjiaowan (−84.3%), and Leidashih (−95.6%) (Table S3). In addition, foliose corals decreased after the 2016 typhoon season (2.6 ± 2.3% in April 2016 and 0.8 ± 0.8% in October 2016). However, by April 2017 they were already showing signs of recovery (1.6 ± 1.7%, *p*< 0.001, KW-test, Dunn test; Fig. 6).

#### Bleaching impacts

Bleaching was observed during the August 2015, September 2016, and October 2016 surveys, with an average bleaching in the benthic communities of 1.6 ± 1.2%. Bleaching peaked in October 2016 with 7.4% of the scleractinian and octocoral assemblages that were affected (2.8% of the benthic communities). Of the 38 OTUs (Table 4 and Table S4) that bleached in 2015–2016, 31 were scleractinian OTUs—16 encrusting, six branching, seven massive, one foliose, and one tabulate coral OTUs. In addition, five additional bleached OTUs were octocorals, one was a branching hydrozoan OTU and one was a sea anemone OTU.

**Table 4:** Spatial repartition of bleached colonies in KNP. Distribution of the bleached colonies in each area and site in KNP. In addition, the numbers of OTUs observed with at least one bleached colony during the 2015–2016 period are reported in the first column of the table, while the percentage of bleached colonies per site compared to the bleached percentage recorded in the respective areas of KNP are reported in the last columns of the table.

		**Bleached OTUs (38)**	**Percentage of total bleached OTUs**	**Percentage of the observed bleached OTUs within each area**
**East Coast**		4	1.6	
	Chufengbi	0	0	0
	Jialeshui	4	1.0	64.0
	Longkeng	1	0.6	36.0
**Nanwan**		33	74.0	
	Houbihu	10	11.8	15.9
	Leidashih	8	21.7	29.4
	Outlet	22	22.6	30.6
	Sangjiaowan	10	14.0	18.9
	Tiaoshih	11	3.9	5.3
**West Coast**		19	24.4	
	Dabaisha	5	5.4	22.3
	Houwan	13	13.2	54.2
	Wanlitung	9	5.8	23.6

**Figure 7:**
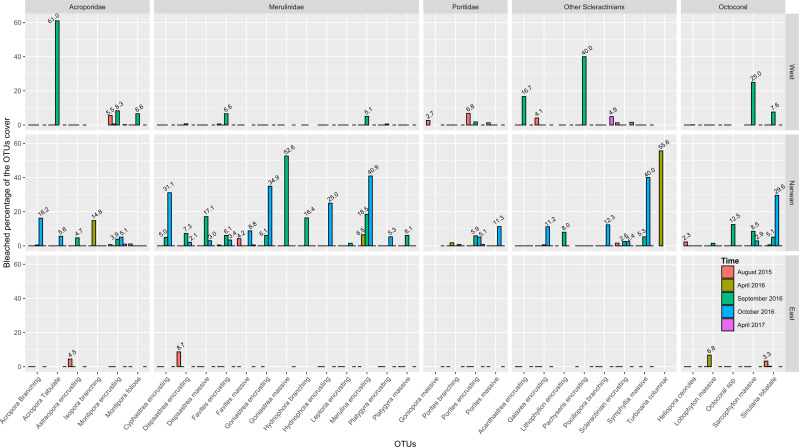
Proportions of bleached scleractinian and octocoral cover throughout KNP. Percentages of bleached cover above 2% were included in the plot.

Despite being the OTU constituting 30.7% of all the bleaching observations from 2015 to 2017, the percentage of bleached encrusting *Montipora* never exceeded 10% of its cover during any survey and for any of the three surveyed regions within KNP (Fig. 7; Table S4). Similarly, the encrusting *Porites, Favites* and *Dipsastraea* were also among the OTUs with the most observations of bleaching (respectively, 7.3%, 5.0% and 2.2% of all the bleaching observations from 2015 to 2017), and yet, the percentage of bleaching cover of each of these three encrusting OTUs never exceeded 10% for any region during any survey (Fig. 7; Table S4). While *Sinularia* represented 22.1% of the bleaching observations, it did suffer more abundantly from bleaching, especially in Nanwan Bay in October 2016 when 29.6% of its cover had bleached (Fig. 7; Table S4). In addition, branching *Acropora* and massive *Porites* (respectively, 3.0% and 2.9% of all the bleaching observations) were noticeably locally impacted by the bleaching in October 2016, with their respective cover being bleached at 16.2% and 11.3% within Nanwan Bay (Fig. 7; Table S4). On the other hand, despite representing only 4.4% of the bleaching observations in KNP, the rare tabulate *Acropora* had been heavily impacted on the West coast, as 61% of its cover had bleached in September 2016 (Fig. 7; Table S4). Similarly, the *Sarcophyton* OTU, the encrusting *Cyphrastrea*, *Goniastrea*, *Hydnophora*, *Merulina* and *Pachyseris* OTUs, along with the massive *Goniastrea* and *Symphyllia* OTUs were heavily impacted by bleaching. Despite representing only a small fraction of the bleaching observations in KNP (≤2%), in terms of total cover, the bleaching level for these OTUs was ≥25% during the September–October 2016 surveys (Fig. 7; Table S4). Geographically, most of the bleached colonies were found within Nanwan (74.0% of the observed colonies; Table 4, Fig. 7). Bleaching was present throughout the bay, but it was highest in Leidashih and Outlet, representing 59.9% of the total bleaching observed within the bay and 44.4% of the total in KNP during our survey period (Table 4). The west coast represented 24.4% of the observed bleached colonies between August 2015–October 2016, with Houwan—near the Baolishi river estuary—representing the most impacted site on the west coast of KNP. Indeed, Houwan represented 54.2% of the bleaching observed on the west coast of KNP and 13.2% of the entire KNP (Table 4). On the other hand, the east coast of KNP represented only 1.6% of the observed bleaching, with Jialeshui representing 64.0% of the east coast bleaching, and only 1.0% of KNP as a whole (Table 4, Fig. 7).

## Discussion

Here, we provide insights into environmental conditions and dynamics of the benthic communities of KNP from August 2015 to April 2017, during which time several typhoons and heat stress events occurred. Benthic data from both before and after disturbances were compared to determine what caused the changes during this time. In 2016, seawater temperatures in KNP only exceeded the locally estimated bleaching threshold (30.1 °C) for a short period of time, contrary to NOAA’s regional estimations. Maximum DHW _L_ estimated for KNP only reached 3.1 °C weeks before the super typhoon Meranti hit Kenting in September 2016 and reduced the heat stress. Successive storms further helped cool waters and limit the impacts of this El Niño event on coral assemblages in southern Taiwan. During the 2016 hot season, an average of 2.2% of KNP benthic communities were bleached. An increase in the frequency and intensity of storms in the region could transform benthic communities, providing (at least temporarily) a different direction from communities shaped by bleaching events. Overall, our results draw attention to the regions with intense storm activity and the role they might play as a refuge area for some reef taxa.

### Heat stress and storms in southern Taiwan

*In situ* seawater temperatures recorded in KNP were slightly lower in the east coast than in the two other areas of KNP. The highest recorded seawater temperature was 31.2 °C, which is 1.1 °C higher than the locally estimated bleaching threshold (30.1 °C) and 1.6 °C higher than the bleaching threshold estimated by NOAA for southern Taiwan (29.6 °C). Respectively, DHW_L_remained ≥1 °C weeks in Houwan and Houbihu for ten and eight weeks, reaching the maximum observed DHW_L_ of 3.1 °C weeks in September 2016 (Houwan). However, major bleaching events have been previously observed at similar DHW levels as the ones observed in our study, in others regions such as the Caribbean (van Hooidonk & Huber, 2009a) and more recently in the Red Sea (Monroe et al., 2018). On the other hand, a study from Paparella et al. (2019) in the Persian Gulf showed that wind events with a speed ≥4 m s^−1^ are sufficient to induce seawater cooling effects, with the intensity of the water cooling being correlated to the wind speed.

In addition, numerous studies have discussed the relationship between typhoons and seawater temperature cooling along the storm track (Lin et al., 2003; Walker, Leben & Balasubramanian, 2005; Zheng & Tang, 2007; White et al., 2013; Paparella et al., 2019; Burt et al., 2019). In the South China Sea, decreases in seawater temperatures by as much as 11 °C were recorded after a category 4 (Saffir-Simpson) typhoon (Shang et al., 2008), with an average seawater temperature drop of 5.1 °C over areas as large as 150,000 km^2^. In Taiwan, over the last two decades, 43 typhoons have induced seawater temperature drops greater than 2 °C (Doong, Peng & Babanin, 2019). Recently in Taiwan, Yang et al. (2019) also showed the influence of typhoons on the rapidity at which the seawater temperature drops can occur and the depth to which the seawater column can be mixed, as evidenced in the 2016 super typhoon Nepartak which led to a 1.5 °C drop within four hours, and mixed the water layers up to 120 m in depth (Yang et al., 2019). In KNP, decreases in seawater temperatures were also observed during or immediately after five of the seven typhoons that occurred between August 2015 and April 2017. Even if the observed seawater temperature changes were small, they were enough to drop or remain below the local bleaching threshold. This effect added to those of the successive typhoons in the region (e.g., in September 2016, there were two super typhoons and one category 4 typhoon within three weeks).

From a more general point of view, DHW_L_ accumulation showed high disparity within KNP (Fig. 3), highlighting the importance of the local environmental context in limiting the heat stress, a factor that NOAA’s regional estimations for southern Taiwan did not consider. Indeed, despite using a 5 km grid, NOAA’s Coral Reef Watch combined under a single DHW estimation for the Southern Taiwan virtual station: subtropical regions within the Taiwan Strait (Penghu Archipelago, Chiayi coastlines), eastern tropical offshore islets (Lanyu and Ludao) and the coastal area ranging from the southern tip of Taiwan moving over 250 km north to Hualien county. In KNP, like the nearby Okinawa Island (Sakai, Singh & Iguchi, 2019) which was hit by at least two typhoons during each summer season in 2015 and 2016, the regional estimates of DHW by NOAA were about three times higher than the *in situ* estimations. Satellite-based Sea Surface Temperature (SSTs) estimations can be crucial for large-scale studies or locations that are difficult to access (Van Hooidonk & Huber, 2009a; Van Hooidonk & Huber, 2009b; Burt et al., 2019). However, the specific environmental context of certain areas—such as KNP, where tide-associated upwelling and recurrent typhoons induced important smaller scale seawater temperature variations (Keshavmurthy et al., 2019; Hsu et al., 2020; Lee et al., 2020)—are not likely to be identified by satellite data; this may have misguided estimates of the capacity of reefs to resist large-scale heat-stresses.

### Impact of typhoons on benthic composition

Typhoons induce changes in KNP’s benthic composition. The cover of massive coral increased after the 2016 typhoon season, which in the long-term could lead to a shift toward higher contribution of mechanical damage-resistant corals in KNP. Consistent with previous studies in other locations (Harmelin-Vivien, 1994; Fabricius et al., 2008; White et al., 2013; Harii et al., 2014), the branching, foliose, and tabulate coral morphotypes in this study were among the most vulnerable to mechanical damages. Similar to what was observed on Ishigaki Island (Okinawa, Japan) (Harii et al., 2014), the low covers of branching, foliose, and tabulate corals observed within KNP could already be a consequence of past typhoons (Puotinen et al., 2020). To reinforce this hypothesis, in a previous study in Taiwan (Ribas-Deulofeu et al., 2016), branching *Acropora* (type *A. muricata*) and foliose *Turbinaria* were found more abundantly in the subtropical Penghu Archipelago (located in the Taiwan strait, and more sheltered from being directly hit by typhoons than Taiwan mainland and its eastern islets, Fig. 1) than in the more tropical and reefal Taiwanese regions such as the eastern island of Ludao (Fig. 1) and KNP, itself. However, in the present study, after a few months, foliose corals showed signs of recovery from mechanical damages throughout KNP—i.e., there were no further significant differences between the initial and final surveys (Fig. 6)—and their distribution slightly increased on the west coast, which is more sheltered from storm-induced mechanical damage than Nanwan (Dai, 1991). Branching, foliose, and tabulate corals give reefs structural complexity, allowing the reefs to offer shelter and food to a wide diversity of organisms (Chong-Seng et al., 2012). Increases in the frequency and/or intensity of typhoons in the coming decades may increase the rate at which those key functional groups are lost (Puotinen et al., 2020), and could even imperil the ecosystem’s ability to maintain itself (Cheal et al., 2017).

Average macro-algae cover increased by 60% between April 2016 (11.4 ± 12.1%) and April 2017 (18.3 ± 13.3%). After the first three typhoons of 2016 hit Taiwan, average macro-algae cover was <2% and increased to >15% by April 2017, six months after the last typhoon of the previous typhoon season hit Taiwan. Macro-algae patterns in Nanwan are characteristic of typhoon-induced changes: a major decrease during the typhoon season as a consequence of typhoons’ wave actions and currents uprooting, breaking, and burying algae (Harmelin-Vivien, 1994), followed by a large increase in the months after the disturbances because of subsequent runoff and land-based pollution (Harmelin-Vivien, 1994; Zhang et al., 2013). In the weeks following typhoons, high nutrient levels trigger algal blooms, which colonize the substrate that was left bare after the storms (Harmelin-Vivien, 1994; Doropoulos et al., 2014), inducing short- to long-term shifts toward a macro-algal state (Roff et al., 2015). Those dynamics further highlight the potential importance of herbivorous organisms in recovering from typhoon impacts (Bozec et al., 2019) and the necessity to adequately manage fish stocks.

### Extents of bleaching

During the 2015 and 2016 summer seasons, on average less than 2% of the KNP benthic community was impacted by bleaching. This percentage peaked at 2.8% in October 2016, when 7.4% of the scleractinian and octocoral assemblages were affected. Overall, scleractinian only showed a slight decline in live cover while octocoral covers did not show any significant decline throughout our survey period, even though the bleaching event in KNP was reported to be severe in a global review of the third global mass bleaching event (Hughes et al., 2018a). In comparison, a study from Hughes et al. (2017b) estimated that, during the 2016 bleaching event, 91.1% of the reefs in GBR were bleached, with almost 50% of the reefs presenting a bleaching level over 60% of their coral cover. Over the following six months, 30% of GBR’s live coral cover was lost and 51% was lost one year after the bleaching event (Stuart-Smith et al., 2018). In the Indian Ocean, the Maldivian reefs lost 75% of their coral cover due to the extensive 2016 bleaching event in the area (Perry & Morgan, 2017).

Within KNP, an important spatial heterogeneity in the bleaching distribution was observed. Despite the cooling effect of the tidal upwelling within the bay (Hsu et al., 2020; Lee et al., 2020), the observed bleaching in 2016 was more substantial in Nanwan compared to the west and east coasts of the Hengchun Peninsula. However, the peak of the bleaching in the west and east coasts of KNP might have been missed as surveys were unable to be conducted due to sea conditions on the west coast in October 2016 and on the east coast in September and October 2016. Furthermore, parameters such as rainfall and consequent water runoff or waves and water flow (McClanahan et al., 2007; Williams et al., 2010; Hongo & Yamano, 2013) might partly contribute to the spatial heterogeneity observed in bleaching intensity and should be differentiated by regions in future studies. The species that were sensitive to bleaching in KNP were similar to those in other locations around the world (Marshall & Baird, 2000; Hongo & Yamano, 2013). Octocorals (24.5%), especially *Sinularia* spp. (22.1%), were highly sensitive to heat stress, as previously documented in various studies (Williams & Bunkley-Williams, 1990; Glynn, 1996; Marshall & Baird, 2000; Floros, Samways & Armstrong, 2004). Among hard corals, branching and tabulate *Acropora* suffered severely from bleaching, which is consistent with a previous definition of bleaching susceptibility in scleractinian corals (Williams & Bunkley-Williams, 1990; Brown & Suharsono, 1990; Glynn, 1996; Marshall & Baird, 2000; Hughes et al., 2017b). However, unlike those reported in the GBR (Marshall & Baird, 2000) and on Okinawa Island (Hongo & Yamano, 2013), encrusting corals such as *Cyphrastrea, Goniastrea, Hydnophora, Merulina* and *Pachyseris* in KNP were among the OTUs most impacted by the bleaching.

Previous bleaching events around the world have been shown to induce shifts in coral assemblages. Certain coral morphotypes, such as branching and tabulate, are considered to be particularly sensitive to heat stress (Marshall & Baird, 2000), and are also the most sensitive morphotypes to mechanical damages (Harmelin-Vivien, 1994; Fabricius et al., 2008). In addition, those groups seem to exhibit low recovery capacities to bleaching, as shown by the successive surveys by Brown & Suharsono (1990) and Warwick, Clarke & Suharsono (1990) on Tikus Island, Indonesia. Years after the perturbations, the coral assemblage shifted toward encrusting and massive morphotypes after major declines in tabulate and branching corals, particularly *Acropora*, *Pocillopora,* and *Montipora* (Denis et al., 2017). Recent studies have found the same pattern after the more recent 2016 bleaching event; for example, in the GBR, the 2016 bleaching event induced shifts in the coral assemblage from being dominated by fast-growing branching and tabular species toward slower-growing and simpler morphological groups such as encrusting and massive corals (Hughes et al., 2018b). Similar phenomena may have taken place in Kenting: the low cover of bleaching-sensitive families such as Acroporidae and Pocilloporidae might be a result of previous bleaching events in KNP, such as the 1998 mass bleaching event (Dai et al., 1999). Annual typhoon disturbances are also likely to be major factors contributing to the low cover of these coral morphotypes in KNP benthic communities.

KNP escaped major bleaching events during the 2015–2016 El Niño cycle better than most of the other coral reef regions around the world (Kayanne, Suzuki & Liu, 2017; Perry & Morgan, 2017; Hughes et al., 2017b; Hughes et al., 2018a; Monroe et al., 2018). *In situ* data showed that decreases in seawater temperatures near the bleaching threshold correspond to the arrival of typhoons around Taiwan. Similar to what was observed in Mauritius during the 1998 worldwide bleaching event (Turner et al., 2000) and events in the Caribbean in 2005 and 2010 (Carrigan & Puotinen, 2014), KNP benefited from tropical storms during the third worldwide mass-bleaching event. Furthermore, the latter study showed that the negative effects of typhoons on the reefs, like mechanical damage, are five times lower than the positive effects resulting from the typhoon-associated cold-wakes (Carrigan & Puotinen, 2014). However, with the current global warming trend and the increase in bleaching event frequency, it is unlikely that the positive effects of typhoons in Taiwan could durably mitigate the impacts of back-to-back bleaching events. However, storms in the Kuroshio region may offer a delay before the bleaching events’ frequency and/or intensity outpace the positive effects of typhoon-induced heat stress releases, therefore constituting a temporary thermal refuge for some reef taxa in this highly diversified area.

## Conclusions

From August 2015 to April 2017, KNP seawater temperatures reached 31.2 °C; the DHW _L_ peaked at 3.1 °C weeks (September 2016) and remained at this level for a week. During our study period, seven typhoon events occurred in Taiwan. Consecutive storms kept thermal stress low and likely played an important role in mitigating the impacts of the 2015–2016 El Niño event. Indeed, KNP did not experience extensive bleaching like most of the world’s coral reefs. Typhoons were shown to offer temporary thermal relief that could have benefited reef organisms on the edge of bleaching. On the other hand, local shifts were observed in the benthic composition that could be related to mechanical damage caused by storm disturbances. Several coral morphologies—e.g., branching, foliose, and tabulate—are uncommon in reefs of southern Taiwan, which could be a long-term consequence of typhoons in the region decreasing their structural complexity and diversity. Overall, our results suggest that greater attention should be paid to those regions with intense storm activity, as they may play a role as resistance refuge areas for at least some reef taxa. Information on regions such as Taiwan, with both a very active typhoon regime and rapidly warming seawater, are essential for the scientific community to better understand the synergetic effects of climate change-associated threats. Furthermore, Taiwanese coral reef responses to those disturbances are particularly important as Taiwan is positioned at a key location between the Coral Triangle region and the potential high latitude refuges in the north-west Pacific Ocean. Filling regional knowledge gaps in Taiwan is crucial to better understand the regional disparities in coral reef assemblages in the face of climate change and how its associated global threats are likely to reshape coral reef ecosystems as well as their distribution in the near future.

##  Supplemental Information

10.7717/peerj.11744/supp-1 Supplemental Information 1 Supplementary Tables

10.7717/peerj.11744/supp-2 Supplemental Information 2 In situ SST and DHW

10.7717/peerj.11744/supp-3 Supplemental Information 3 Benthic communities raw dataset

## Additional Information and Declarations

### Data Availability

The following information was supplied regarding data availability: Benthic communities raw data are available in the Supplemental Files.

## References

[ref-1] Arnholt AT, Evans B (2017). https://CRAN.R-project.org/package=BSDA.

[ref-2] Belkin IM (2009). Rapid warming of large marine ecosystems. Progress in Oceanography.

[ref-3] Borcard D, Gillet F, Legendre P (2018). Numerical ecology with R.

[ref-4] Bozec YM, Doropoulos C, Roff G, Mumby PJ (2019). Transient grazing and the dynamics of an unanticipated coral–algal phase shift. Ecosystems.

[ref-5] Brown BE, Suharsono (1990). Damage and recovery of coral reefs affected by El Niño related seawater warming in the Thousand Islands, Indonesia. Coral Reefs.

[ref-6] Burt JA, Paparella F, Al-Mansoori N, Al-Mansoori A, Al-Jailani H (2019). Causes and consequences of the 2017 coral bleaching event in the southern Persian/Arabian Gulf. Coral Reefs.

[ref-7] Carballo-Bolaños R, Denis V, Huang YY, Keshavmurthy S, Chen CA (2019). Temporal variation and photochemical efficiency of species in Symbiodinaceae associated with coral Leptoria phrygia (Scleractinia; Merulinidae) exposed to contrasting temperature regimes. PLOS ONE.

[ref-8] Carilli JE, Norris RD, Black BA, Walsh SM, McField M (2009). Local stressors reduce coral resilience to bleaching. PLOS ONE.

[ref-9] Carrigan AD, Puotinen M (2014). Tropical cyclone cooling combats region-wide coral bleaching. Global Change Biology.

[ref-10] Cheal AJ, MacNeil MA, Emslie MJ, Sweatman H (2017). The threat to coral reefs from more intense cyclones under climate change. Global Change Biology.

[ref-11] Chen CC, Shiah FK, Lee HJ, Li KY, Meng PJ, Kao SJ, Tseng YF, Chung CL (2005). Phytoplankton and bacterioplankton biomass, production and turnover in a semi-enclosed embayment with spring tide induced upwelling. Marine Ecology Progress Series.

[ref-12] Chong-Seng KM, Mannering TD, Pratchett MS, Bellwood DR, Graham NAJ (2012). The influence of coral reef benthic condition on associated fish assemblages. PLOS ONE.

[ref-13] Dai C-F (1991). Reef environment and coral fauna of southern Taiwan. Atoll Research Bulletin.

[ref-14] Dai C-F, Horng S (2009a). Scleractinia fauna of Taiwan I. The complex group.

[ref-15] Dai C-F, Horng S (2009b). Scleractinia fauna of Taiwan II. The robust group.

[ref-16] Dai C-F, Kuo KM, Chen YT, Chuang CH (1999). Changes of coral communities on the east and west coast of the Kenting National Park. Journal of National Park (in Chinese).

[ref-17] Denis V, Ribas-Deulofeu L, Sturaro N, Kuo CY, Chen CA (2017). A functional approach to the structural complexity of coral assemblages based on colony morphological features. Scientific Reports.

[ref-18] Denis V, Soto D, De Palmas S, Lin YTV, Benayahu Y, Huang YM, Liu SL, Chen JW, Chen Q, Sturaro N, Ho MJ, Su Y, Dai C-F, Chen CA, Loya Y, Puglise KA, Bridge TCL (2019). Taiwan. Mesophotic coral ecosystems.

[ref-19] Dinno A, Dinno M (2017). http://cran.stat.unipd.it/web/packages/dunn.test/dunn.test.pdf.

[ref-20] Doong DJ, Peng JP, Babanin AV (2019). Field investigations of coastal sea surface temperature drop after typhoon passages. Earth System Science Data.

[ref-21] Doropoulos C, Roff G, Zupan M, Nestor V, Isechal AL, Mumby PJ (2014). Reef-scale failure of coral settlement following typhoon disturbance and macroalgal bloom in Palau, Western Pacific. Coral Reefs.

[ref-22] Eakin CM, Lough JM, Heron SF, Liu G (2009). Climate variability and change: monitoring data and evidence for increased coral bleaching stress. Coral Bleaching: patterns, processes, causes and consequences. Ecological Studies.

[ref-23] Eakin CM, Sweatman HPA, Brainard RE (2019). The 2014–2017 global-scale coral bleaching event: insights and impacts. Coral Reefs.

[ref-24] Edmunds PJ (2017). Unusually high coral recruitment during the 2016 El Niño in Mo’orea, French Polynesia. PLOS ONE.

[ref-25] Fabricius K, Alderslade P (2001). Soft corals and sea fans: a comprehensive guide to the tropical shallow water genera of the central-west Pacific, the Indian Ocean and the Red Sea.

[ref-26] Fabricius KE, De’ath G, Puotinen ML, Done T, Cooper TF, Burgess SC (2008). Disturbance gradients on inshore and offshore coral reefs caused by a severe tropical cyclone. Limnology and Oceanography.

[ref-27] Floros CD, Samways MJ, Armstrong B (2004). Taxonomic patterns of bleaching within a South African coral assemblage. Biodiversity and Conservation.

[ref-28] Glynn PW (1996). Coral reef bleaching: facts, hypotheses and implications. Global Change Biology.

[ref-29] Goreau TJ, Hayes RL (1994). Coral bleaching and ocean hot spots. Ambio.

[ref-30] Goreau T, McClanahan T, Hayes R, Strong A (2000). Conservation of Coral Reefs after the 1998 Global Bleaching Event. Conservation Biology.

[ref-31] Harii S, Hongo C, Ishihara M, Ide Y, Kayanne H (2014). Impacts of multiple disturbances on coral communities at Ishigaki Island, Okinawa, Japan, during a 15 year survey. Marine Ecology Progress Series.

[ref-32] Harley CDG, Hughes AR, Hultgren KM, Miner BG, Sorte CJB, Thornber CS, Rodriguez LF, Tomanek L, Williams SL (2006). The impacts of climate change in coastal marine systems. Ecology Letters.

[ref-33] Harmelin-Vivien ML (1994). The effects of storms on coral reefs: a review. Journal of Coastal Research.

[ref-34] Hervé M (2019). https://cran.r-project.org/web/packages/RVAideMemoire/RVAideMemoire.pdf.

[ref-35] Hoegh-Guldberg O (1999). Climate change, coral bleaching and the future of the world’s coral reefs. Marine and Freshwater Research.

[ref-36] Hongo C, Yamano H (2013). Species-specific responses of corals to bleaching events on anthropogenically turbid reefs on Okinawa Island, Japan, over a 15-year period (1995–2009). PLOS ONE.

[ref-37] Hsieh TC, Ma KH, Chao A (2016). iNEXT: an R package for rarefaction and extrapolation of species diversity (Hill numbers). Methods in Ecology and Evolution.

[ref-38] Hsu PC, Lee HJ, Zheng Q, Lai JW, Su FC, Ho CR (2020). Tide-induced periodic sea surface temperature drops in the coral reef area of Nanwan Bay, southern Taiwan. Journal of Geophysical Research: Oceans.

[ref-39] Huang CC, Hung TC, Fan KL (1991). Nonbiological factors associating with coral bleaching events in the shallow water near the outlet of the third nuclear power plant in Southern Taiwan. Acta Oceanographica Taiwanica.

[ref-40] Hughes TP, Anderson KD, Connolly SR, Heron SF, Kerry JT, Lough JM, Baird AH, Baum JK, Berumen ML, Bridge TC, Claar DC, Eakin CM, Gilmour JP, Graham NAJ, Harrison H, Hobbs JPA, Hoey AS, Hoogenboom M, Lowe RJ, McCulloch MT, Pandolfi JM, Pratchett M, Schoepf V, Torda G, Wilson SK (2018a). Spatial and temporal patterns of mass bleaching of corals in the Anthropocene. Science.

[ref-41] Hughes TP, Barnes ML, Bellwood DR, Cinner JE, Cumming GS, Jackson JBC, Kleypas J, VandeLeemput IA, Lough JM, Morrison TH, Palumbi SR, VanNes EH, Scheffer M (2017a). Coral reefs in the Anthropocene. Nature.

[ref-42] Hughes TP, Kerry JT, Baird AH, Connolly SR, Dietzel A, Eakin CM, Heron SF, Hoey AS, Hoogenboom MO, Liu G, McWilliam MJ, Pears RJ, Pratchett MS, Skirving WJ, Stella JS, Torda G (2018b). Global warming transforms coral reef assemblages. Nature.

[ref-43] Hughes TP, Kerry JT, Álvarez Noriega M, Álvarez Romero JG, Anderson KD, Baird AH, Babcock RC, Beger M, Bellwood DR, Berkelmans R, Bridge TC, Butler IR, Byrne M, Cantin NE, Comeau S, Connolly SR, Cumming GS, Dalton SJ, Diaz-Pulido G, Eakin CM, Figueira WF, Gilmour JP, Harrison HB, Heron SF, Hoey AS, Hobbs JPA, Hoogenboom MO, Kennedy EV, Kuo C, Lough JM, Lowe RJ, Liu G, McCulloch MT, Malcolm HA, McWilliam MJ, Pandolfi JM, Pears RJ, Pratchett MS, Schoepf V, Simpson T, Skirving WJ, Sommer B, Torda G, Wachenfeld DR, Willis BL, Wilson SK (2017b). Global warming and recurrent mass bleaching of corals. Nature.

[ref-44] Jan S, Chen CTA (2009). Potential biogeochemical effects from vigorous internal tides generated in Luzon Strait: a case study at the southernmost coast of Taiwan. Journal of Geophysical Research: Oceans.

[ref-45] Jiang JJ, Lee CL, Fang MDer, Tu BW, Liang YJ (2015). Impacts of emerging contaminants on surrounding aquatic environment from a youth festival. Environmental Science and Technology.

[ref-46] Kayanne H, Suzuki R, Liu G (2017). Bleaching in the Ryukyu Islands in 2016 and associated Degree Heating Week threshold, Galaxea. Journal of Coral Reef Studies.

[ref-47] Keshavmurthy S, Kuo CY, Huang YY, Carballo-Bolaños R, Meng P, Wang JT, Chen CA (2019). Coral Reef Resilience in Taiwan: lessons from Long-Term Ecological Research on the Coral Reefs of Kenting National Park (Taiwan). Journal of Marine Science and Engineering.

[ref-48] Kohler KE, Gill SM (2006). Coral Point Count with Excel extensions (CPCe): a visual basic program for the determination of coral and substrate coverage using random point count methodology. Computers & Geosciences.

[ref-49] Kuo CY, Meng PJ, Ho PH, Wang JT, Chen JP, Chiu YW, Lin HJ, Chang Y-C, Fan T, Chen CA (2011). Damage to the reefs of Siangjiao Bay marine protected area of Kenting National Park, Southern Taiwan during typhoon Morakot. Zoological Studies.

[ref-50] Kuo CY, Yuen YS, Meng PJ, Ho PH, Wang JT, Liu PJ, Chang Y-C, Dai C-F, Fan T, Lin HJ, Baird AH, Chen CA (2012). Recurrent disturbances and the degradation of hard coral communities in Taiwan. PLOS ONE.

[ref-51] Lee H, Chao S, Fan K, Kuo T (1999). Tide-induced eddies and upwelling in a semi-enclosed basin: Nan Wan. Estuarine, Coastal and Shelf Science.

[ref-52] Lee HJ, Chao SY, Fan KL, Wang YH, Liang NK (1997). Tidally induced upwelling in a semi-enclosed basin: Nan Wan Bay. Journal of Oceanography.

[ref-53] Lee IH, Fan TY, Fu KH, Ko DS (2020). Temporal variation in daily temperature minima in coral reefs of Nanwan Bay, Southern Taiwan. Scientific Reports.

[ref-54] Lee CS, Huang LR, Shen HS, Wang ST (2006). A climatology model for forecasting typhoon rainfall in Taiwan. Natural Hazards.

[ref-55] Lejeusne C, Chevaldonné P, Pergent-Martini C, Boudouresque CF, Pérez T (2010). Climate change effects on a miniature ocean: the highly diverse, highly impacted Mediterranean Sea. Trends in Ecology and Evolution.

[ref-56] Lin I, Liu WT, Wu CC, Wong GTF, Hu C, Chen Z, Liang WDer, Yang Y, Liu KK (2003). New evidence for enhanced ocean primary production triggered by tropical cyclone. Geophysical Research Letters.

[ref-57] Lin YV, Denis V (2019). Acknowledging differences: number, characteristics, and distribution of marine benthic communities along Taiwan coast. Ecosphere.

[ref-58] Liu G, Heron SF, Mark Eakin C, Muller-Karger FE, Vega-Rodriguez M, Guild LS, De la Cour JL, Geiger EF, Skirving WJ, Burgess TFR, Strong AE, Harris A, Maturi E, Ignatov A, Sapper J, Li J, Lynds S (2014). Reef-scale thermal stress monitoring of coral ecosystems: new 5-km global products from NOAA coral reef watch. Remote Sensing.

[ref-59] Liu G, Strong AE, Skirving W (2003). Remote sensing of sea surface temperatures during 2002 barrier reef coral bleaching. Eos, Transactions American Geophysical Union.

[ref-60] Liu PJ, Meng PJ, Liu LL, Wang JT, Leu MY (2012). Impacts of human activities on coral reef ecosystems of southern Taiwan: a long-term study. Marine Pollution Bulletin.

[ref-61] Liu PJ, Shao KT, Jan RQ, Fan T, Wong SL, Hwang JS, Chen JP, Chen CC, Lin HJ (2009). A trophic model of fringing coral reefs in Nanwan Bay, southern Taiwan suggests overfishing. Marine Environmental Research.

[ref-62] Manzello DP, Brandt M, Smith TB, Lirman D, Hendee JC, Nemeth RS (2007). Hurricanes benefit bleached corals. Proceedings of the National Academy of Sciences of the United States of America.

[ref-63] Marshall PA, Baird AH (2000). Bleaching of corals on the Great Barrier Reef: Differential susceptibilities among taxa. Coral Reefs.

[ref-64] Maynard N, Château P-A, Ribas-Deulofeu L, Liou JL (2019). Using internet surveys to estimate visitors’ willingness to pay for coral reef conservation in the Kenting National Park, Taiwan. Water.

[ref-65] McClanahan TR, Ateweberhan M, Muhando CA, Maina J, Mohammed MS (2007). Effects of climate and seawater temperature variation on coral bleaching and mortality. Ecological Monographs.

[ref-66] McCune B, Grace J (2002). PC-ORD analysis of ecological communities.

[ref-67] Meng PJ, Lee HJ, Wang JT, Chen CC, Lin HJ, Tew KS, Hsieh WJ (2008). A long-term survey on anthropogenic impacts to the water quality of coral reefs, southern Taiwan. Environmental Pollution.

[ref-68] Monroe AA, Ziegler M, Roik A, Röthig T, Hardenstine RS, Emms MA, Jensen T, Voolstra CR, Berumen ML (2018). In situ observations of coral bleaching in the central Saudi Arabian Red Sea during the 2015/2016 global coral bleaching event. PLOS ONE.

[ref-69] Montgomery RS, Strong AE (1994). Coral bleaching threatens oceans, life. Eos, Transactions American Geophysical Union.

[ref-70] Moritz C, Vii J, Lee Long W, Jerker T, Thomassin A, Planes S (2018). Status and trends of coral reefs of the Pacific.

[ref-71] Oksanen J (2018). Vegan: ecological diversity.

[ref-72] Paparella F, Xu C, Vaughan GO, Burt JA (2019). Coral bleaching in the Persian/Arabian Gulf is modulated by summer winds. Frontiers in Marine Science.

[ref-73] Pecl GT, Araújo MB, Bell JD, Blanchard J, Bonebrake TC, Chen IC, Clark TD, Colwell RK, Danielsen F, Evengård B, Falconi L, Ferrier S, Frusher S, Garcia RA, Griffis RB, Hobday AJ, Janion-Scheepers C, Jarzyna MA, Jennings S, Lenoir J, Linnetved HI, Martin VY, McCormack PC, McDonald J, Mitchell NJ, Mustonen T, Pandolfi JM, Pettorelli N, Popova E, Robinson SA, Scheffers BR, Shaw JD, Sorte CJB, Strugnell JM, Sunday JM, Tuanmu MN, Vergés A, Villanueva C, Wernberg T, Wapstra E, Williams SE (2017). Biodiversity redistribution under climate change: impacts on ecosystems and human well-being. Science.

[ref-74] Perry C, Morgan K (2017). Bleaching drives collapse in reef carbonate budgets and reef growth potential on southern Maldives reefs. Scientific Reports.

[ref-75] Puotinen ML, Drost E, Lowe R, Depczynski M, Radford B, Heyward A, Gilmour J (2020). Towards modelling the future risk of cyclone wave damage to the world’s coral reefs. Global Change Biology.

[ref-76] Ribas-Deulofeu L, Denis V, De Palmas S, Kuo CY, Hsieh HJ, Chen CA (2016). Structure of benthic communities along the Taiwan latitudinal gradient. PLOS ONE.

[ref-77] Rivest EB, Gouhier TC (2015). Complex environmental forcing across the biogeographical range of coral populations. PLOS ONE.

[ref-78] Roff G, Doropoulos C, Zupan M, Rogers A, Steneck RS, Golbuu Y, Mumby PJ (2015). Phase shift facilitation following cyclone disturbance on coral reefs. Oecologia.

[ref-79] Sakai K, Singh T, Iguchi A (2019). Bleaching and post-bleaching mortality of *Acropora* corals on a heat-susceptible reef in 2016. PeerJ.

[ref-80] Shang S, Li L, Sun F, Wu J, Hu C, Chen D, Ning X, Qiu Y, Zhang C, Shang S (2008). Changes of temperature and bio-optical properties in the South China Sea in response to Typhoon Lingling, 2001. Geophysical Research Letters.

[ref-81] Spalding M, Burke L, Wood SA, Ashpole J, Hutchison J, Ermgassen Pzu (2017). Mapping the global value and distribution of coral reef tourism. Marine Policy.

[ref-82] Strong AE, Barrientos CS, Duda C (1996). Improved satellite techniques for monitoring coral reef bleaching. Proceedings of the 8th International Coral Reef Symposium.

[ref-83] Stuart-Smith RD, Brown CJ, Ceccarelli DM, Edgar GJ (2018). Ecosystem restructuring along the Great Barrier Reef following mass coral bleaching. Nature.

[ref-84] Sully S, Burkepile DE, Donovan MK, Hodgson G, Van Woesik R (2019). A global analysis of coral bleaching over the past two decades. Nature Communications.

[ref-85] Teh LSL, Teh LCL, Sumaila UR (2013). A global estimate of the number of coral reef fishers. PLOS ONE.

[ref-86] Tew KS, Leu MY, Wang JT, Chang CM, Chen CC, Meng PJ (2014). A continuous, real-time water quality monitoring system for the coral reef ecosystems of Nanwan Bay, Southern Taiwan. Marine Pollution Bulletin.

[ref-87] Titlyanov E, Titlyanov T (2012). Marine plants of the Asian Pacific Region countries, their use and cultivation.

[ref-88] Toda M, Watanabe M (2020). Mechanisms of enhanced ocean surface warming in the Kuroshio region for 1951–2010. Climate Dynamics.

[ref-89] Tu JY, Chou C, Chu PS (2009). The abrupt shift of typhoon activity in the vicinity of Taiwan and its association with Western North Pacific–East Asian climate change. Journal of Climate.

[ref-90] Tung CP, Tseng CH, Dai C-F, Lee PF (2007). Early warning indicators and framework of climate change to ecosystem sustainability in Taiwan.

[ref-91] Turner J, Hardman E, Klaus R, Fagoonee I, Daby D, Baghooli R, Persands S (2000). The Reefs of Mauritius. Coral reef degradation in the Indian Ocean: status report 2000.

[ref-92] Van Hooidonk R, Huber M (2009a). Quantifying the quality of coral bleaching predictions. Coral Reefs.

[ref-93] Van Hooidonk R, Huber M (2009b). Equivocal evidence for a thermostat and unusually low levels of coral bleaching in the Western Pacific Warm Pool. Geophysical Research Letters.

[ref-94] Vergés A, Steinberg PD, Hay ME, Poore AGB, Campbell AH, Ballesteros E, Heck KL, Booth DJ, Coleman MA, Feary DA, Figueira W, Langlois T, Marzinelli EM, Mizerek T, Mumby PJ, Nakamura Y, Roughan M, Van Sebille E, Sen GuptaA, Smale DA, Tomas F, Wernberg T, Wilson SK (2014). The tropicalization of temperate marine ecosystems: : climate-mediated changes in herbivory and community phase shifts. Proceedings of the Royal Society B: Biological Sciences.

[ref-95] Veron JEN (2000). Corals of the World.

[ref-96] Walker ND, Leben RR, Balasubramanian S (2005). Hurricane-forced upwelling and chlorophyll a enhancement within cold-core cyclones in the Gulf of Mexico. Geophysical Research Letters.

[ref-97] Warwick RM, Clarke KR, Suharsono P (1990). A statistical analysis of coral community responses to the 1982-83 El Niño in the Thousand Islands, Indonesia. Coral Reefs.

[ref-98] White KN, Ohara T, Fujii T, Kawamura I, Mizuyama M, Montenegro J, Shikiba H, Naruse T, McClelland T, Denis V, Reimer JD (2013). Typhoon damage on a shallow mesophotic reef in Okinawa, Japan. PeerJ.

[ref-99] White KN, Weinstein DK, Ohara T, Denis V, Montenegro J, Reimer JD (2017). Shifting communities after–typhoon damage on an upper mesophotic reef in Okinawa, Japan. PeerJ.

[ref-100] Wild C, Hoegh-Guldberg O, Naumann MS, Colombo-Pallotta MF, Ateweberhan M, Fitt WK, Iglesias-Prieto R, Palmer C, Bythell JC, Ortiz JC, Loya Y, Van Woesik R (2011). Climate change impedes scleractinian corals as primary reef ecosystem engineers. Marine and Freshwater Research.

[ref-101] Wilkinson CR, Souter D (2008). Status of Caribbean coral reefs after bleaching and hurricanes in 2005.

[ref-102] Williams EH, Bunkley-Williams L (1990). The worldwide coral reef bleaching cycle and related sources of coral mortality. Atoll Research Bulletin.

[ref-103] Williams GJ, Knapp IS, Maragos JE, Davy SK (2010). Modeling patterns of coral bleaching at a remote Central Pacific atoll. Marine Pollution Bulletin.

[ref-104] Yang YJ, Chang MH, Hsieh CY, Chang HI, Jan S, Wei CL (2019). The role of enhanced velocity shears in rapid ocean cooling during super typhoon Nepartak 2016. Nature Communications.

[ref-105] Zhang J, Wang DR, Jennerjahn T, Dsikowitzky L (2013). Land-sea interactions at the east coast of Hainan Island, South China Sea: a synthesis. Continental Shelf Research.

[ref-106] Zheng G, Tang D (2007). Offshore and nearshore chlorophyll increases induced by typhoon winds and subsequent terrestrial rainwater runoff. Marine Ecology Progress Series.

