# Supplementary material for: Impacts of heat stress and storm events on the benthic communities of Kenting National Park (Taiwan)
Source: PeerJ. 2021 Jul 23;9:e11744. doi: 10.7717/peerj.11744 (PMC8312492; doi:10.7717/peerj.11744)
Supplement: Supplemental Information 1 [file peerj-09-11744-s001.pdf]

Impacts of heat stress and storm events on the benthic communities of Kenting National Park (Taiwan)

Lauriane Ribas-Deulofeu[1,2,3], Vianney Denis[4], Pierre-Alexandre Château[5], Chaolun Allen Chen[1,2,3,6*]

[1]Biodiversity Research Center, Academia Sinica, Nangang, Taipei 115, Taiwan

[2]Taiwan International Graduate Program-Biodiversity, Academia Sinica, Nangang, Taipei 115, Taiwan

[3]Department of Life Science, National Taiwan Normal University, Taipei 106, Taiwan

[4]Institute of Oceanography, National Taiwan University, Taipei 106, Taiwan

[5]Department of Marine Environment and Engineering, National Sun Yat-sen University, Kaohsiung 804, Taiwan

[6]Department of Life Science, Tunghai University, Taichung 404, Taiwan

*Corresponding author:

Chaolun Allen Chen, Biodiversity Research Center, Academia Sinica, Nangang, Taipei, Taiwan 115. E-mail: cac@gate.sinica.edu.tw;

Tel: +886-2-2789-9549

Supplementary Tables

Table S1. Source of environmental datasets. (Websites accessed: 5 June 2021).

Variables	Stations	GPS coordinates	Time	Source
Rainfall and Wind Speed	C0R350	21.9217°N, 120.7358°E	August 2nd, 2015 to April 24th, 2017	Central Weather Bureau http://e-service.cwb.gov.tw/HistoryDataQuery
	C0R360	21.9456°N, 120.8022°E		
	C0R370	21.9919°N, 120.8445°E		
	C0R620	21.9008°N, 120.8552°E		
	C1R320	22.0744°N, 120.7162°E		
Wave Height	Eluanbi buoy	21.9006°N, 120.8314°E	August 2nd, 2015 to April 24th, 2017	Central Region Water Resources Office, Water Resources Agency, Ministry of Economic Affairs https://eng.wra.gov.tw/
Long term SST	Houbihu buoy	21.9459°N, 120.7453°E	January 1st, 2007 to December 31st, 2014	Central Weather Bureau http://www.cwb.gov.tw
in situ Seawater Temperature	Jialeshui 5m	21.9923°N, 120.8635°E	August 2nd, 2015 to April 24th, 2017	CREEG lab (Supplementary File S1)
	Houwan 5m	22.0441°N, 120.6943°E	August 2nd, 2015 to April 24th, 2017	
	Houbihu 5m	21.9429°N, 120.9429°E	August 2nd, 2015 to October 23rd, 2016	

Table S2. KNP benthic composition in average percentage of cover from August 2015–April 2017. Average percent of cover in the east coast (Tables A1 and A2), Nanwan (Tables B1 and B2), and the west coast (Tables C1 and C2). Standard deviations are in parentheses.

**A1. Distribution of the first seven major categories**		Ascidian	Hydrozoa	Algae			Octocoral	Scleractinian
				ECA	Macro-algae	Turf		
East coast								
Chufengbi (120.8973, 22.09112)	August 2015	0.0 (0.0)	0.0 (0.0)	13.2 (2.7)	13.8 (10.0)	57.9 (15.2)	7.5 (3.1)	0.6 (0.5)
	April 2016	0.0 (0.0)	0.1 (0.2)	6.7 (2.3)	23.4 (11.1)	56.8 (16.3)	6.1 (2.6)	1.0 (0.8)
	September 2016	NA	NA	NA	NA	NA	NA	NA
	October 2016	NA	NA	NA	NA	NA	NA	NA
	April 2017	0.0 (0.0)	0.0 (0.0)	7.4 (3.3)	11.1 (4.6)	71.8 (5.7)	4.7 (2.2)	0.3 (0.5)
Jialeshui (120.8636, 21.99232)	August 2015	0.0 (0.0)	0.9 (0.5)	9.5 (5.9)	11.2 (5.2)	60.0 (9.0)	3.7 (5.1)	11.0 (1.7)
	April 2016	0.2 (0.3)	2.7 (3.2)	8.2 (4.3)	35.5 (11.0)	37.5 (7.3)	4.8 (4.1)	5.8 (4.4)
	September 2016	NA	NA	NA	NA	NA	NA	NA
	October 2016	NA	NA	NA	NA	NA	NA	NA
	April 2017	0.02 (0.1)	1.6 (2.0)	10.1 (3.5)	40.2 (7.0)	38.9 (8.2)	0.6 (0.5)	2.0 (1.3)
Longkeng (120.8619, 21.91283)	August 2015	0.0 (0.0)	2.6 (2.8)	12.4 (2.5)	2.8 (1.7)	44.8 (7.4)	18.8 (7.3)	12.3 (5.4)
	April 2016	0.1 (0.2)	3.5 (2.3)	17.5 (3.5)	10.3 (7.0)	33.1 (5.8)	12.1 (7.8)	16.2 (4.3)
	September 2016	NA	NA	NA	NA	NA	NA	NA
	October 2016	NA	NA	NA	NA	NA	NA	NA
	April 2017	0.0 (0.0)	0.6 (1.0)	22.3 (10.4)	10.0 (5.9)	34.5 (15.8)	18.2 (9.1)	10.1 (4.8)

**A2. Distribution of the last seven major categories**		Sea anemone	Sponge	Zoantharian	Other life	Unknown	Substrate	
							Unstable Substrate	Bare Substrate
East Coast								
Chufengbi (120.8973, 22.09112)	August 2015	0.0 (0.0)	0.0 (0.0)	0.0 (0.0)	0.7 (1.2)	0.0 (0.0)	6.3 (3.6)	0.0 (0.0)
	April 2016	0.0 (0.0)	0.0 (0.0)	0.0 (0.0)	0.3 (0.7)	0.0 (0.0)	5.4 (4.5)	0.3 (0.2)
	September 2016	NA	NA	NA	NA	NA	NA	NA
	October 2016	NA	NA	NA	NA	NA	NA	NA
	April 2017	0.0 (0.0)	0.0 (0.0)	0.0 (0.0)	0.3 (0.7)	0.0 (0.0)	4.3 (2.3)	0.1 (0.1)
Jialeshui (120.8636, 21.99232)	August 2015	0.0 (0.0)	0.0 (0.0)	0.0 (0.1)	0.8 (0.5)	0.0 (0.0)	2.4 (3.3)	0.5 (0.6)
	April 2016	0.0 (0.0)	0.1 (0.1)	0.0 (0.0)	0.0 (0.0)	0.0 (0.0)	0.2 (0.2)	5.0 (2.2)
	September 2016	NA	NA	NA	NA	NA	NA	NA
	October 2016	NA	NA	NA	NA	NA	NA	NA
	April 2017	0.0 (0.0)	0.0 (0.0)	0.1 (0.2)	0.16 (0.2)	0.0 (0.0)	2.5 (3.5)	3.9 (5.7)
Longkeng (120.8619, 21.91283)	August 2015	0.0 (0.0)	0.0 (0.0)	0.7 (0.2)	1.4 (0.4)	0.0 (0.0)	0.5 (0.6)	3.7 (1.4)
	April 2016	0.0 (0.0)	0.1 (0.1)	0.3 (0.4)	1.0 (0.8)	0.0 (0.0)	0.5 (0.7)	5.4 (1.7)
	September 2016	NA	NA	NA	NA	NA	NA	NA
	October 2016	NA	NA	NA	NA	NA	NA	NA
	April 2017	0.0 (0.0)	0.5 (0.6)	0.2 (0.2)	0.3 (0.5)	0.0 (0.0)	0.6 (0.4)	2.8 (0.7)

B1. Distribution of the first seven major categories		Ascidian	Hydrozoa	Algae			Octocoral	Scleractinian
				ECA	Macro-algae	Turf		
Nanwan								
Houbihu (120.7518, 21.94299)	August 2015	0.0 (0.0)	5.5 (4.5)	3.8 (2.7)	1.9 (1.8)	30.3 (4.9)	8.8 (1.0)	33.0 (6.0)
	April 2016	0.0 (0.0)	1.4 (1.3)	5.3 (2.2)	1.4 (1.0)	29.3 (9.3)	13.6 (10.8)	39.0 (11.7)
	September 2016	0.0 (0.0)	1.0 (1.1)	3.9 (1.3)	0.5 (0.5)	25.8 (5.3)	10.8 (2.4)	38.9 (13.9)
	October 2016	0.0 (0.0)	0.6 (0.6)	6.3 (1.1)	1.2 (0.7)	31.1 (7.0)	4.7 (1.9)	33.3 (13.2)
	April 2017	0.0 (0.0)	1.0 (0.9)	7.7 (1.7)	4.4 (3.2)	23.9 (2.3)	9.6 (4.2)	37.1 (2.1)
Leidashih (120.7448, 21.93034)	August 2015	0.0 (0.0)	3.6 (2.1)	2.3 (1.1)	1.6 (1.0)	57.8 (2.8)	8.8 (5.5)	24.0 (5.6)
	April 2016	0.0 (0.0)	3.5 (5.0)	8.87 (3.5)	16.9 (7.7)	39.1 (9.2)	11.6 (8.5)	16.9 (6.8)
	September 2016	0.0 (0.0)	0.5 (0.8)	5.4 (3.0)	0.1 (0.2)	52.1 (15.8)	22.0 (17.4)	6.2 (3.4)
	October 2016	0.0 (0.0)	0.3 (0.3)	7.5 (3.3)	0.4 (0.2)	64.4 (10.7)	18.0 (15.3)	8.6 (5.2)
	April 2017	0.0 (0.0)	0.3 (0.3)	3.2 (1.6)	28.2 (9.7)	30.5 (6.2)	29.3 (9.8)	5.1 (3.8)
Outlet (120.7446, 21.93137)	August 2015	0.0 (0.0)	3.1 (2.3)	1.3 (0.9)	2.7 (2.1)	43.1 (12.4)	3.7 (2.7)	43.5 (11.6)
	April 2016	0.0 (0.0)	3.0 (1.9)	11.1 (5.2)	7.4 (3.7)	21.4 (10.1)	6.7 (3.3)	38.9 (9.1)
	September 2016	0.0 (0.0)	5.9 (7.3)	5.8 (2.1)	2.4 (1.5)	30.3 (5.45)	1.23 (0.8)	43.06 (4.19)
	October 2016	0.0 (0.0)	7.4 (8.9)	5.1 (2.2)	2.2 (2.0)	28.3 (4.0)	2.8 (2.4)	45.0 (10.0)
	April 2017	0.0 (0.0)	3.8 (3.5)	8.1 (3.6)	10.2 (3.3)	24.7 (7.7)	1.5 (1.2)	43.6 (7.4)
Sangjiaowan (120.8296, 21.92399)	August 2015	0.0 (0.0)	0.2 (0.3)	7.6 (2.3)	3.7 (0.7)	44.0 (4.6)	1.4 (0.6)	38.7 (6.1)
	April 2016	0.0 (0.0)	0.1 (0.2)	8.9 (4.4)	8.2 (12.7)	36.1 (10.0)	3.6 (2.6)	39.4 (22.3)
	September 2016	0.0 (0.0)	0.1 (0.2)	5.6 (2.8)	1.0 (0.5)	26.7 (2.7)	2.4 (1.9)	42.3 (8.3)
	October 2016	0.0 (0.0)	0.4 (0.8)	9.5 (3.9)	0.6 (0.5)	27.5 (9.6)	0.6 (0.6)	39.4 (7.4)
	April 2017	0.0 (0.0)	0.1 (0.1)	4.6 (2.0)	39.4 (2.5)	24.4 (3.5)	0.8 (0.3)	21.8 (2.8)
Tiaoshih (120.7707, 21.95193)	August 2015	0.0 (0.1)	2.4 (1.3)	10.1 (3.9)	3.4 (1.9)	35.3 (10.1)	2.0 (1.2)	36.3 (9.6)
	April 2016	0.0 (0.0)	2.6 (1.1)	2.7 (2.2)	9.1 (10.3)	27.3 (6.2)	3.1 (1.5)	42.53 (16.0)
	September 2016	0.0 (0.0)	1.4 (1.0)	5.5 (1.3)	0.9 (0.3)	27.4 (4.6)	2.2 (0.8)	29.0 (7.0)
	October 2016	0.0 (0.0)	1.5 (1.2)	6.1 (1.6)	0.9 (0.5)	47.1 (8.8)	1.6 (0.8)	28.3 (5.5)
	April 2017	0.0 (0.0)	1.5 (0.8)	5.2 (1.2)	33.1 (11.4)	16.0 (2.9)	1.6 (0.6)	25.8 (9.7)

**B2. Distribution of the last seven major categories**		Sea anemone	Sponge	Zoantharian	Other life	Unknown	Substrate	
							Unstable Substrate	Bare Substrate
Nanwan								
Houbihu (120.7518, 21.94299)	August 2015	0.0 (0.0)	0.2 (0.2)	0.1 (0.1)	0.1 (0.1)	0.0 (0.0)	15.5 (3.3)	0.9 (0.6)
	April 2016	0.0 (0.0)	0.4 (0.4)	0.1 (0.1)	0.3 (0.5)	0.0 (0.0)	7.9 (3.4)	1.4 (0.8)
	September 2016	0.2 (0.4)	0.3 (0.4)	0.1 (0.2)	0.4 (0.5)	0.0 (0.0)	11.1 (6.4)	7.1 (4.3)
	October 2016	0.0 (0.0)	0.0 (0.1)	0.2 (0.1)	0.2 (0.1)	0.0 (0.0)	17.6 (6.7)	4.7 (3.9)
	April 2017	0.0 (0.0)	0.4 (0.8)	0.1 (0.1)	0.1 (0.3)	0.0 (0.0)	11.3 (6.9)	4.5 (1.2)
Leidashih (120.7448, 21.93034)	August 2015	0.0 (0.0)	0.0 (0.0)	0.2 (0.2)	0.0 (0.1)	0.0 (0.0)	0.9 (0.7)	0.8 (0.6)
	April 2016	0.0 (0.0)	0.1 (0.0)	0.4 (0.5)	0.5 (0.3)	0.0 (0.0)	0.8 (0.6)	1.3 (0.5)
	September 2016	0.0 (0.0)	0.0 (0.1)	0.0 (0.0)	0.1 (0.1)	0.0 (0.0)	7.4 (5.3)	6.3 (5.7)
	October 2016	0.0 (0.0)	0.0 (0.0)	0.1 (0.3)	0.0 (0.0)	0.0 (0.0)	0.6 (0.4)	0.2 (0.2)
	April 2017	0.0 (0.0)	0.0 (0.0)	0.1 (0.1)	0.2 (0.2)	0.0 (0.0)	2.0 (0.9)	1.0 (0.8)
Outlet (120.7446, 21.93137)	August 2015	0.0 (0.0)	0.1 (0.2)	0.1 (0.1)	0.1 (0.1)	0.0 (0.0)	0.4 (0.4)	2.1 (1.3)
	April 2016	0.0 (0.0)	0.4 (0.4)	0.3 (0.6)	0.1 (0.2)	0.0 (0.0)	7.3 (7.3)	3.5 (1.0)
	September 2016	0.0 (0.0)	0.0 (0.0)	0.2 (0.4)	0.1 (0.1)	0.0 (0.0)	3.5 (3.2)	7.6 (1.0)
	October 2016	0.0 (0.0)	0.1 (0.3)	0.2 (0.2)	0.0 (0.1)	0.2 (0.0)	2.3 (3.7)	6.6 (7.2)
	April 2017	0.0 (0.0)	0.1 (0.1)	0.0 (0.0)	0.0 (0.1)	0.0 (0.1)	4.4 (4.4)	3.6 (2.9)
Sangjiaowan (120.8296, 21.92399)	August 2015	0.0 (0.0)	0.1 (0.1)	0.2 (0.3)	0.2 (0.2)	0.0 (0.0)	3.3 (1.4)	0.7 (0.9)
	April 2016	0.0 (0.0)	0.2 (0.2)	0.0 (0.0)	0.4 (0.6)	0.0 (0.0)	0.7 (0.9)	2.5 (2.7)
	September 2016	0.0 (0.0)	0.2 (0.4)	0.0 (0.0)	0.1 (0.1)	0.0 (0.0)	14.9 (4.2)	6.8 (5.1)
	October 2016	0.0 (0.0)	0.0 (0.0)	0.0 (0.0)	0.5 (0.4)	0.0 (0.0)	3.8 (2.8)	17.8 (8.7)
	April 2017	0.0 (0.0)	0.0 (0.1)	0.0 (0.0)	0.2 (0.1)	0.0 (0.0)	7.1 (1.9)	1.6 (1.2)
Tiaoshih (120.7707, 21.95193)	August 2015	0.0 (0.1)	0.5 (05)	0.1 (0.2)	0.8 (0.4)	0.1 (0.1)	8.6 (4.6)	0.4 (0.4)
	April 2016	0.0 (0.0)	0.9 (0.6)	0.2 (0.3)	0.4 (0.2)	0.0 (0.0)	9.3 (4.1)	1.8 (1.3)
	September 2016	0.0 (0.0)	0.5 (0.3)	0.1 (0.2)	0.3 (0.2)	0.0 (0.0)	19.6 (2.4)	13.0 (8.6)
	October 2016	0.0 (0.0)	0.5 (0.1)	0.0 (0.1)	0.6 (0.5)	0.0 (0.0)	11.3 (5.7)	2.1 (1.3)
	April 2017	0.0 (0.0)	0.0 (0.0)	0.1 (0.1)	0.3 (0.3)	0.0 (0.0)	13.9 (3.8)	2.5 (0.5)

**C1. Distribution of the first seven major categories**		Ascidian	Hydrozoa	Algae			Octocoral	Scleractinian
				ECA	Macro-algae	Turf		
West Coast								
Dabaisha (120.7137, 21.93376)	August 2015	0.0 (0.0)	0.9 (1.2)	3.7 (3.7)	1.5 (0.9)	63.0 (0.9)	6.6 (6.2)	21.0 (3.8)
	April 2016	0.0 (0.0)	0.2 (0.2)	10.3 (5.2)	6.8 (2.9)	38.1 (8.4)	4.1 (3.5)	34.3 (6.1)
	September 2016	0.0 (0.0)	0.2 (0.3)	6.9 (2.3)	2.8 (1.3)	34.3 (5.8)	10.6 (5.3)	34.3 (1.8)
	October 2016	NA	NA	NA	NA	NA	NA	NA
	April 2017	0.0 (0.0)	0.4 (0.3)	6.7 (2.7)	5.5 (1.1)	32.5 (5.5)	5.9 (4.7)	39.1 (6.7)
Houwan (120.6943, 22.04417)	August 2015	0.0 (0.0)	0.1 (0.2)	18.9 (3.3)	0.1 (0.1)	16.6 (5.6)	4.3 (5.1)	50.1 (11.8)
	April 2016	0.0 (0.0)	0.4 (0.5)	5.8 (2.2)	2.6 (4.2)	26.2 (11.1)	7.6 (4.2)	46.6 (12.7)
	September 2016	0.0 (0.0)	0.2 (0.3)	9.2 (3.7)	0.1 (0.1)	22.8 (14.9)	9.9 (8.4)	47.4 (9.0)
	October 2016	NA	NA	NA	NA	NA	NA	NA
	April 2017	0.0 (0.0)	0.5 (0.4)	2.0 (2.2)	14.8 (13.9)	30.4 (11.4)	4.2 (5.0)	34.9 (15.5)
Wanlitung (120.7021, 21.99557)	August 2015	0.0 (0.0)	0.1 (0.3)	9.0 (4.3)	4.2 (2.2)	55.1 (4.4)	4.4 (2.2)	16.9 (5.3)
	April 2016	0.0 (0.0)	0.5 (0.4)	4.4 (2.4)	4.2 (2.5)	57.8 (4.2)	3.8 (1.9)	17.5 (3.3)
	September 2016	0.0 (0.0)	0.1 (0.1)	3.2 (1.9)	1.5 (1.0)	61.2 (9.7)	5.0 (2.5)	19.9 (7.4)
	October 2016	NA	NA	NA	NA	NA	NA	NA
	April 2017	0.0 (0.0)	0.3 (0.4)	7.1 (2.2)	4.4 (1.2)	45.3 (10.6)	5.4 (1.7)	23.5 (7.2)

**C2. Distribution of the last seven major categories**		Sea anemone	Sponge	Zoantharian	Other life	Unknown	Substrate	
							Unstable Substrate	Bare Substrate
West Coast								
Dabaisha (120.7137, 21.93376)	August 2015	0.0 (0.0)	0.1 (0.2)	0.0 (0.1)	0.2(0.4)	0.0 (0.0)	1.5 (1.6)	1.6 (2.4)
	April 2016	0.0 (0.0)	0.1 (0.2)	0.0 (0.0)	0.3 (0.3)	0.0 (0.0)	4.1 (2.2)	1.9 (1.1)
	September 2016	0.0 (0.0)	0.0 (0.1)	0.2 (0.2)	0.2 (0.2)	0.0 (0.0)	6.3 (2.1)	4.3 (3.3)
	October 2016	NA	NA	NA	NA	NA	NA	NA
	April 2017	0.0 (0.0)	0.4 (0.7)	0.0 (0.0)	0.3 (0.2)	0.0 (0.0)	8.4 (3.0)	0.6 (0.3)
Houwan (120.6943, 22.04417)	August 2015	0.0 (0.0)	0.1 (0.1)	0.1 (0.2)	0.3 (0.2)	0.0 (0.0)	0.8 (1.2)	8.6 (5.2)
	April 2016	0.0 (0.0)	0.1 (0.2)	0.0 (0.0)	0.6 (0.4)	0.0 (0.1)	1.7 (1.9)	8.2 (5.6)
	September 2016	0.0 (0.0)	0.3 (0.5)	0.0 (0.1)	0.5 (0.2)	0.0 (0.0)	0.9 (1.5)	8.7 (4.1)
	October 2016	NA	NA	NA	NA	NA	NA	NA
	April 2017	0.0 (0.0)	0.3 (0.6)	0.1 (0.1)	0.5 (0.5)	0.0 (0.0)	4.2 (2.2)	8.31 (5.7)
Wanlitung (120.7021, 21.99557)	August 2015	0.0 (0.0)	0.1 (0.2)	0.0 (0.1)	0.5 (0.3)	0.0 (0.0)	8.8 (4.4)	0.9 (0.7)
	April 2016	0.0 (0.0)	0.4 (0.5)	0.1 (0.1)	0.6 (0.3)	0.0 (0.0)	9.7 (4.8)	1.1 (0.9)
	September 2016	0.0 (0.0)	0.2 (0.5)	0.1 (0.1)	0.8 (0.4)	0.0 (0.0)	7.4 (3.7)	0.7 (0.5)
	October 2016	NA	NA	NA	NA	NA	NA	NA
	April 2017	0.0 (0.0)	0.0 (0.0)	0.1 (0.1)	0.8 (0.5)	0.0 (0.0)	12.4 (5.5)	0.7 (0.7)

Table S3. Scleractinian distributions in KNP from August 2015–April 2017. Average percentage of scleractinian covers for each morphological group in the east coast (Table A), Nanwan (Table B), and the west coast (Table C). Standard deviations are in parentheses.

**A. Distribution of the different morphologies of scleractinians**		Scleractinian categories				
		Branching corals	Encrusting corals	Foliose corals	Massive corals	Tabulate corals
East Coast						
Chufengbi (120.8973, 22.09112)	August 2015	0.1 (0.1)	0.4 (0.5)	0.0 (0.0)	0.0 (0.0)	0.0 (0.0)
	April 2016	0.0 (0.1)	0.7 (0.4)	0.0 (0.0)	0.2 (0.5)	0.0 (0.0)
	September 2016	NA	NA	NA	NA	NA
	October 2016	NA	NA	NA	NA	NA
	April 2017	0.2 (0.3)	0.1 (0.2)	0.0 (0.0)	0.0 (0.0)	0.0 (0.0)
Jialeshui (120.8636, 21.99232)	August 2015	0.7 (0.4)	9.5 (1.5)	0.1 (0.2)	0.7 (0.9)	0.0 (0.0)
	April 2016	0.5 (0.3)	5.3 (4.4)	0.0 (0.0)	0.0 (0.0)	0.0 (0.0)
	September 2016	NA	NA	NA	NA	NA
	October 2016	NA	NA	NA	NA	NA
	April 2017	0.0 (0.1)	1.4 (0.4)	0.0 (0.0)	0.6 (1.3)	0.0 (0.0)
Longkeng (120.8619, 21.91283)	August 2015	1.0 (0.6)	10.1 (4.5)	0.0 (0.0)	1.1 (0.8)	0.0 (0.0)
	April 2016	1.6 (1.1)	14.5 (4.5)	0.0 (0.0)	0.1 (0.2)	0.0 (0.0)
	September 2016	NA	NA	NA	NA	NA
	October 2016	NA	NA	NA	NA	NA
	April 2017	1.3 (1.5)	7.6 (3.8)	0.0 (0.1)	1.1 (1.1)	0.0 (0.0)

**B. Distribution of the different morphologies of scleractinians**		Scleractinian categories				
		Branching corals	Encrusting corals	Foliose corals	Massive corals	Tabulate corals
Nanwan						
Houbihu (120.7518, 21.94299)	August 2015	5.8 (3.0)	11.9 (1.3)	8.2 (5.1)	7.0 (3.6)	0.1 (0.3)
	April 2016	10.9 (5.7)	17.4 (6.2)	2.5 (2.1)	8.2 (8.4)	0.0 (0.0)
	September 2016	7.7 (6.6)	22.4 (5.2)	0.6 (0.6)	8.2 (7.8)	0.1 (0.2)
	October 2016	8.2 (7.9)	18.1 (5.7)	0.7 (1.0)	6.2 (2.7)	0.2 (0.4)
	April 2017	5.8 (2.6)	20.2 (4.2)	1.4 (1.3)	8.8 (4.5)	1.0 (1.4)
Leidashih (120.7448, 21.93034)	August 2015	2.3 (1.8)	17.0 (3.9)	0.3 (0.2)	4.4 (2.6)	0.0 (0.0)
	April 2016	1.6 (1.2)	13.8 (4.3)	0.1 (0.3)	1.4 (1.4)	0.0 (0.0)
	September 2016	0.0 (0.0)	5.3 (2.7)	0.0 (0.0)	0.9 (0.8)	0.0 (0.0)
	October 2016	0.0 (0.1)	7.5 (4.5)	0.0 (0.0)	1.1 (0.8)	0.0 (0.0)
	April 2017	0.1 (0.3)	4.3 (3.3)	0.0 (0.0)	0.7 (0.9)	0.0 (0.0)
Outlet (120.7446, 21.93137)	August 2015	3.1 (0.9)	25.3 (5.5)	5.79 (4.0)	9.3 (6.5)	0.0 (0.1)
	April 2016	4.0 (1.6)	25.3 (5.4)	6.1 (7.0)	3.5 (2.2)	0.0 (0.0)
	September 2016	1.8 (2.2)	35.3 (4.0)	1.9 (2.2)	3.7 (2.0)	0.4 (0.5)
	October 2016	2.8 (2.2)	34.1 (10.8)	2.0 (1.7)	6.1 (2.3)	0.0 (0.0)
	April 2017	2.3 (1.5)	30.4 (3.0)	4.4 (3.4)	6.6 (6.3)	0.0 (0.0)
Sangjiaowan (120.8296, 21.92399)	August 2015	2.7 (1.1)	29.7 (9.0)	2.4 (2.5)	2.1 (1.8)	1.9 (1.2)
	April 2016	2.0 (1.3)	31.8 (18.2)	3.3 (2.3)	1.0 (1.6)	1.4 (1.5)
	September 2016	2.2 (1.5)	36.7 (9.9)	0.0 (0.0)	0.9 (1.0)	2.5 (2.1)
	October 2016	2.0 (1.8)	34.2 (6.3)	0.1 (0.1)	2.92 (2.59)	0.3 (0.3)
	April 2017	0.4 (0.1)	14.9 (2.4)	0.6 (0.6)	3.7 (2.7)	2.3 (2.8)
Tiaoshih (120.7707, 21.95193)	August 2015	6.5 (1.0)	17.9 (4.3)	1.7 (0.9)	4.2 (3.2)	6.1 (4.4)
	April 2016	7.8 (3.0)	21.4 (6.9)	0.9 (0.9)	0.7 (0.7)	11.8 (8.1)
	September 2016	1.4 (0.6)	21.6 (6.0)	0.1 (0.1)	1.3 (1.1)	4.6 (3.1)
	October 2016	2.7 (1.4)	16.0 (4.0)	1.1 (0.6)	2.3 (1.2)	6.2 (4.0)
	April 2017	1.1 (1.2)	15.0 (5.7)	1.8 (1.8)	6.3 (3.1)	1.6 (1.1)

**C. Distribution of the different morphologies of scleractinians**		Scleractinian categories				
		Branching corals	Encrusting corals	Foliose corals	Massive corals	Tabulate corals
West Coast						
Dabaisha (120.7137, 21.93376)	August 2015	1.9 (1.6)	11.5 (4.6)	2.4 (2.1)	5.2 (2.9)	0.0 (0.0)
	April 2016	4.1 (4.7)	18.4 (11.3)	8.3 (1.8)	3.4 (4.0)	0.1 (0.1)
	September 2016	3.7 (3.7)	25.9 (8.3)	2.4 (2.6)	2.4 (2.8)	0.0 (0.0)
	October 2016	NA	NA	NA	NA	NA
	April 2017	2.2 (1.4)	23.3 (11.5)	8.3 (6.2)	5.3 (3.1)	0.1 (0.3)
Houwan (120.6943, 22.04417)	August 2015	2.8 (1.6)	31.9 (10.8)	2.6 (1.8)	12.1 (4.5)	0.7 (0.5)
	April 2016	1.0 (0.6)	38.9 (10.0)	4.1 (3.1)	1.3 (1.3)	1.3 (1.1)
	September 2016	1.2 (1.0)	40.4 (6.3)	2.5 (1.1)	2.3 (2.5)	1.1 (1.1)
	October 2016	NA	NA	NA	NA	NA
	April 2017	0.8 (0.9)	32.0 (13.8)	1.1 (0.5)	0.8 (1.3)	0.2 (0.3)
Wanlitung (120.7021, 21.99557)	August 2015	1.3 (1.0)	8.0 (1.3)	1.8 (2.1)	5.8 (2.7)	0.1 (0.1)
	April 2016	2.6 (1.8)	10.6 (2.6)	0.9 (0.9)	3.1 (3.5)	0.3 (0.5)
	September 2016	1.2 (0.8)	13.2 (1.9)	0.5 (1.2)	4.4 (7.4)	0.5 (1.2)
	October 2016	NA	NA	NA	NA	NA
	April 2017	1.3 (0.6)	15.2 (5.)	3.0 (2.9)	3.4 (1.8)	0.6 (1.0)

Table S4. Proportions of bleached cover (at the OTUs level) within KNP from August 2015 to April 2017. Symbol [/] indicates that the OTUs was not observed during the survey making the bleached percentage irrelevant in these cases.

		Bleached percentages of the OTU's cover														
	Region	East Coast					Nanwan					West Coast				
Family	OTUs	2015-August	2016-April	2016-Sept	2016-Oct	2017-April	2015-August	2016-April	2016-Sept	2016-Oct	2017-April	2015-August	2016-April	2016-Sept	2016-Oct	2017-April
Acroporidae	*Acropora* Branching	0.0	0.0	NA	NA	0.0	0.0	0.0	0.5	16.2	0.0	0.0	0.0	0.0	NA	0.0
	Acropora Tabulate	/	/	NA	NA	0.0	0.0	0.0	0.0	5.6	0.0	0.0	0.0	61.0	NA	0.0
	Astreopora encrusting	4.5	0.0	NA	NA	0.0	0.0	0.0	4.7	0.0	0.0	0.0	0.0	0.0	NA	0.0
	Isopora branching	/	/	NA	NA	/	0.0	14.8	0.0	0.0	0.0	/	/	/	NA	/
	Montipora encrusting	0.0	0.0	NA	NA	0.0	0.7	0.0	3.9	5.1	1.0	5.5	0.6	8.3	NA	0.2
	Montipora foliose	0.0	/	NA	NA	0.0	1.1	0.0	0.0	0.0	0.0	0.0	0.0	6.6	NA	0.0
Merulinidae	*Cyphastrea* encrusting	0.0	0.0	NA	NA	0.0	0.0	0.0	5.0	31.1	0.0	0.0	0.0	0.0	NA	0.0
	Dispsastrea encrusting	8.7	0.0	NA	NA	0.0	0.0	0.0	7.3	2.1	0.0	0.0	0.0	0.7	NA	0.0
	Dispsastrea massive	0.0	0.0	NA	NA	0.0	0.0	0.0	17.1	3.0	0.0	0.0	0.0	0.0	NA	0.0
	Favites encrusting	0.0	0.0	NA	NA	0.0	0.5	0.0	6.1	3.4	0.0	0.6	0.0	6.6	NA	0.0
	Favites massive	0.0	/	NA	NA	0.0	4.2	0.0	0.0	8.8	0.5	0.0	0.0	/	NA	0.0
	Goniastrea encrusting	/	0.0	NA	NA	/	0.0	0.0	6.1	34.9	0.0	0.0	0.0	0.0	NA	0.0
	Goniastrea massive	/	/	NA	NA	0.0	0.0	0.0	52.6	0.0	0.0	0.0	0.0	/	NA	0.0
	Hydnophora branching	/	/	NA	NA	/	0.0	0.0	16.4	0.0	0.0	/	0.0	0.0	NA	/
	Hydnophora encrusting	0.0	0.0	NA	NA	0.0	0.0	0.0	0.0	25.0	0.0	0.0	0.0	0.0	NA	0.0
	Leptoria encrusting	0.0	0.0	NA	NA	0.0	0.0	0.0	0.0	1.5	0.0	0.0	0.0	0.0	NA	0.0
	Merulina encrusting	0.0	/	NA	NA	/	0.0	6.5	18.5	40.9	0.0	0.0	0.0	5.1	NA	0.0
	Platygyra encrusting	0.0	0.0	NA	NA	0.0	0.0	0.0	0.0	5.3	0.0	0.0	0.0	0.6	NA	0.0
	Platygyra massive	0.0	0.0	NA	NA	0.0	0.0	0.0	6.1	0.0	0.0	0.0	0.0	0.0	NA	0.0
Poritidae	*Goniopora* massive	/	/	NA	NA	0.0	/	/	/	/	0.0	2.7	/	/	NA	0.0
	Porites branching	0.0	/	NA	NA	0.0	0.0	1.8	0.0	0.8	0.0	0.0	0.0	0.0	NA	0.0
	Porites encrusting	0.0	0.0	NA	NA	0.0	0.0	0.0	5.9	5.1	0.8	6.8	0.0	1.8	NA	0.0
	Porites massive	0.0	/	NA	NA	0.0	0.0	0.0	0.0	11.3	0.0	1.3	0.0	0.0	NA	0.0
Other Scleractinians	*Acanthastrea* encrusting	0.0	0.0	NA	NA	0.0	0.0	0.0	0.0	0.0	0.0	0.0	0.0	16.7	NA	0.0
	Galaxea encrusting	0.0	0.0	NA	NA	0.0	0.0	0.0	0.6	11.2	0.0	4.1	0.0	0.0	NA	0.0
	Lithophyllon encrusting	/	/	NA	NA	/	0.0	/	8.0	0.0	/	/	0.0	0.0	NA	0.0
	Pachyseris encrusting	0.0	/	NA	NA	0.0	0.0	0.0	0.0	0.0	0.0	0.0	0.0	40.0	NA	0.0
	Pocillopora branching	0.0	0.0	NA	NA	0.0	0.0	0.0	0.0	12.3	0.0	0.0	0.0	0.0	NA	4.9
	Scleractinian encrusting	0.0	0.0	NA	NA	0.0	1.6	0.0	2.6	2.4	0.0	1.3	0.0	0.0	NA	1.6
	Symphyllia massive	/	0.0	NA	NA	0.0	0.0	0.0	5.3	40.0	0.0	0.0	0.0	0.0	NA	0.0
	Turbinaria columnar	/	/	NA	NA	/	/	55.6	/	/	/	/	/	/	NA	/
Octocoral	*Heliopora ceorulea*	0.0	0.0	NA	NA	0.0	2.3	0.0	0.0	0.0	0.0	0.0	0.0	0.1	NA	0.0
	Lobophyton massive	0.0	6.8	NA	NA	0.0	0.0	0.0	1.5	0.0	0.0	0.0	0.0	0.0	NA	0.0

	Sarcophyton massive	0.0	0.0	NA	NA	0.0	0.0	0.0	8.5	2.9	0.0	0.0	0.0	25.0	NA	0.0
	Octocoral spp.	0.0	0.0	NA	NA	0.0	0.0	0.0	12.5	0.0	0.0	0.0	0.0	/	NA	/
	Sinularia lobatate	3.3	0.3	NA	NA	0.0	0.0	0.6	5.1	29.6	0.0	0.0	0.0	7.6	NA	0.0
Hydrozoa	*Millepora* branching	0.0	0.0	NA	NA	0.0	0.3	0.0	0.0	0.0	0.0	0.0	0.0	0.0	NA	0.0
Sea Anemone	Sea Anemone	/	/	NA	NA	/	0.0	/	0.0	/	/	/	/	100.0	NA	/
